# Supplementary material for: Discovery of a selective alpha-kinase 1 inhibitor for the rare genetic disease ROSAH syndrome
Source: Nat Commun. 2025 Sep 9;16:8251. doi: 10.1038/s41467-025-63731-5 (PMC12420824; doi:10.1038/s41467-025-63731-5)
Supplement: Supplementary file 1 — Supplementary Information [file 41467_2025_63731_MOESM1_ESM.pdf]

TITLE

Discovery of a Selective Alpha-Kinase 1 Inhibitor for the Rare Genetic Disease ROSAH Syndrome

SUPPLEMENTARY INFORMATION

Supplementary Figures 1-5

Supplementary Tables 1-3

Supplementary Notes 1-2

Supplementary References

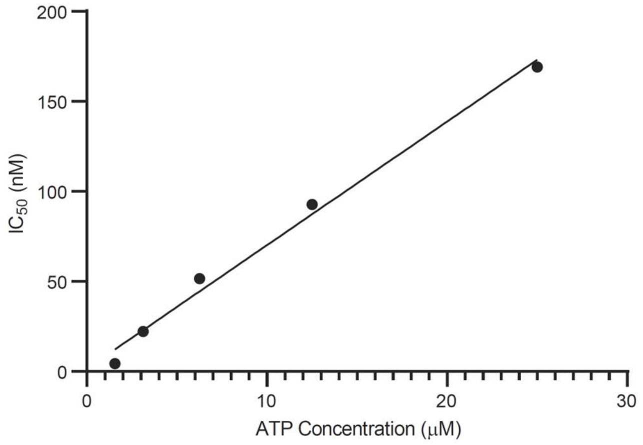

Supplementary Figure 1. DF-003 is an ATP-competitive kinase inhibitor.

The IC_{50} of DF-003 was measured in an HTRF kinase assay with various ATP concentrations.

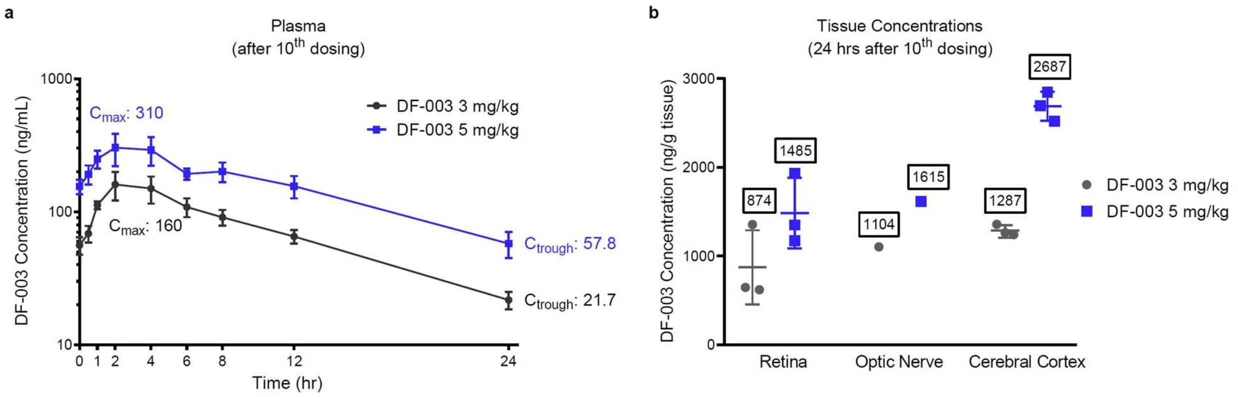

Supplementary Figure 2. Evaluation of the pharmacokinetic properties of DF-003 in mice on day 10 of a repeat daily dosing study. C57BL/6J male mice were orally administered DF-003 at 3 and 5 mg/kg body weight once per day for 10 days (n = 3 animals for each dose). **a** At various time points through 24 h after the last (10th) dose, DF-003 concentrations in the plasma were measured. **b** 24 h after the last (10th) dose, two retinas, two optic nerves, and a sample of the cerebral cortex were collected from each mouse. The two retinas from individual mice were combined into one sample. The six optic nerves from 3 mice in the same dosing group were combined into one sample. Each dot represents a measurement of one sample. DF-003 concentrations in plasma and tissue were determined using LC-MS/MS. Data represent the mean ± SD of the three mice from each dosing group.

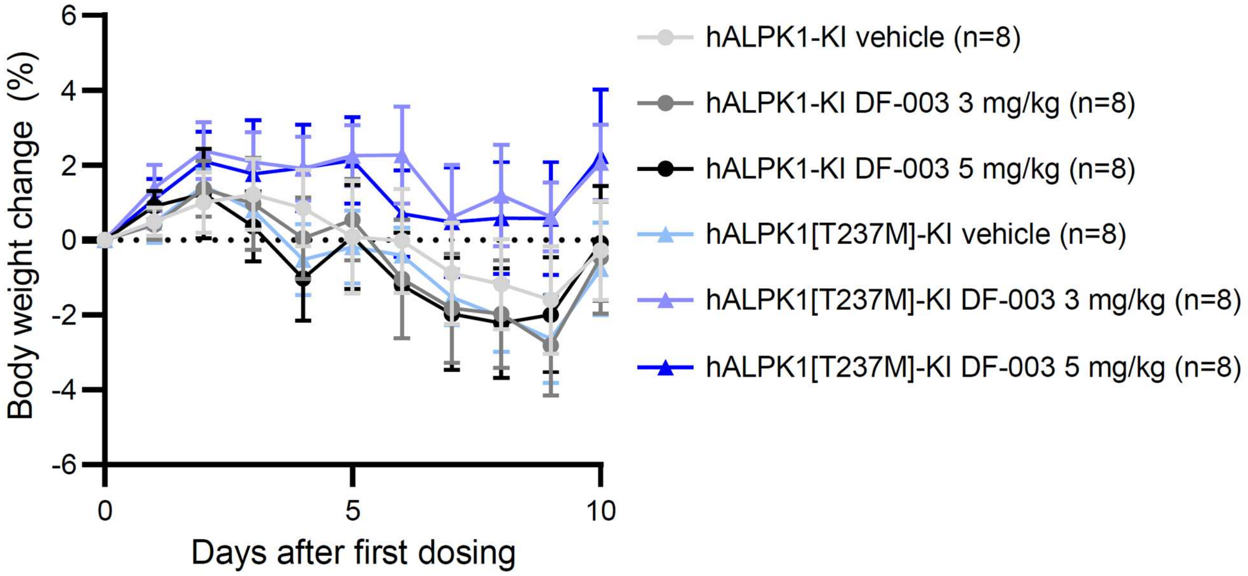

Supplementary Figure 3. DF-003 administration did not cause body weight loss in mice. 16- to 17-week-old female hALPK1-KI or hALPK1[T237M]-KI mice were orally administered with 3 mg/kg or 5 mg/kg DF-003, once a day, for 10 days (n=8 animals). The body weight of each mouse was measured daily immediately before compound injection. Body weight changes compared to the start of the study were represented as % changes. Data represent mean ± SEM for each genotype and treatment group.

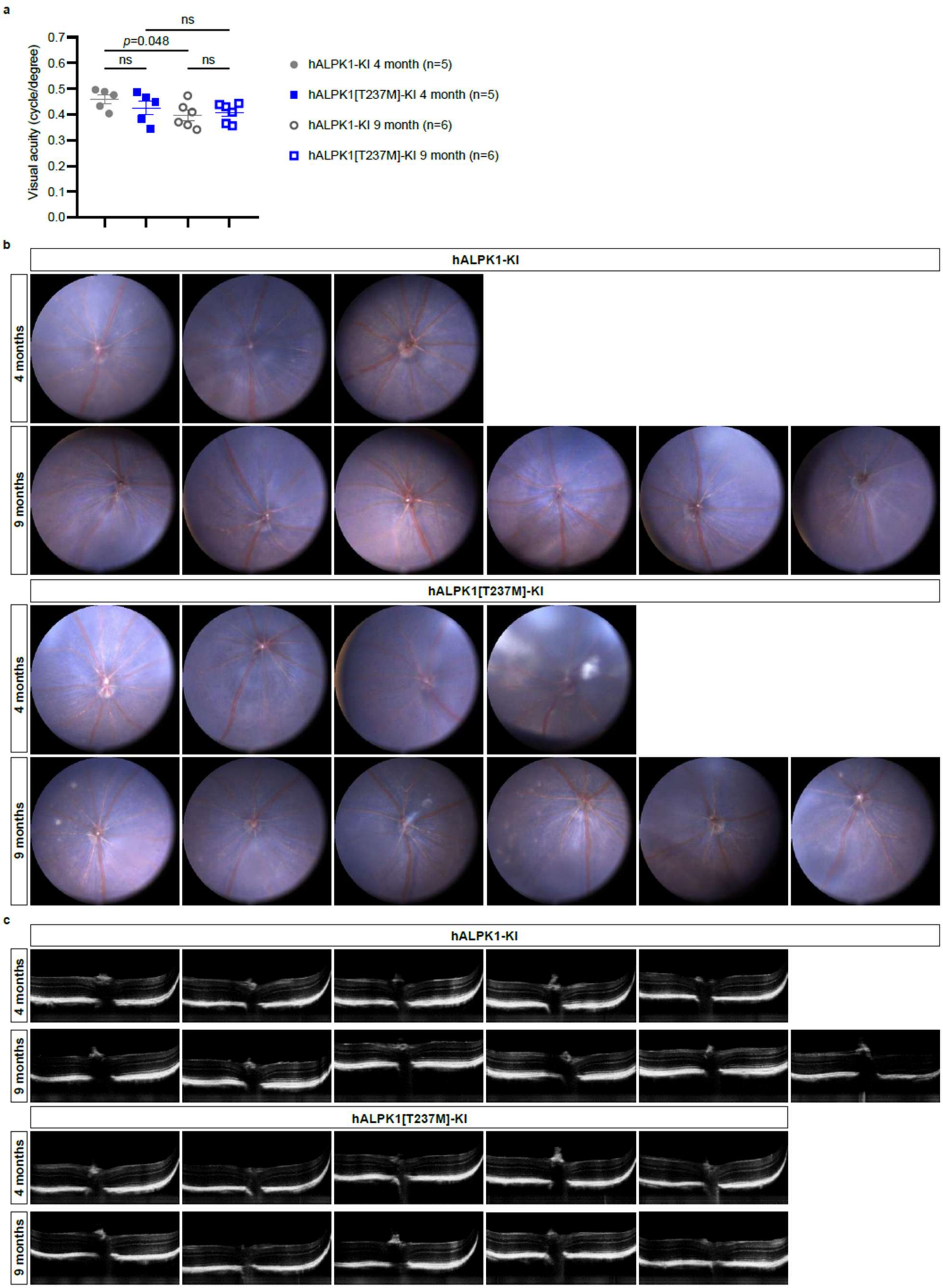

a
ns
p=0.048
ns
ns
Visual acuity (cycle/degree)
0.7
0.6
0.5
0.4
0.3
0.2
0.1
0.0
hALPK1-KI 4 month (n=5)
hALPK1[T237M]-KI 4 month (n=5)
hALPK1-KI 9 month (n=6)
hALPK1[T237M]-KI 9 month (n=6)
b
hALPK1-KI
4 months
9 months
hALPK1[T237M]-KI
4 months
9 months
c
hALPK1-KI
4 months
9 months
hALPK1[T237M]-KI
4 months
9 months

Supplementary Figure 4. hALPK1[T237M]-KI mice do not exhibit visual deficits or retinal degeneration. a hALPK1-KI and hALPK1[T237M]-KI mice underwent visual acuity testing at 4 (5 animals for each genotype) and 9 months (6 animals for each genotype) of age using an OptoDrum system. Each dot represents the visual threshold of one animal. Unpaired, two-tailed Student's t-tests were performed between groups as indicated in the chart. Between 4-month and 9-month-old groups of hALPK1-KI mice, F=1.531, t=2.285, df=9, and p=0.048. **b, c** The same mice underwent fundus imaging of the left eye (**b**), and optical coherence tomography of the right eye (**c**). Images of inadequate quality, any eyes that had developed cataracts due to the anesthesia procedure, and those eyes that failed to be successfully aligned on the acquisition machine after several attempts were excluded from analyses.

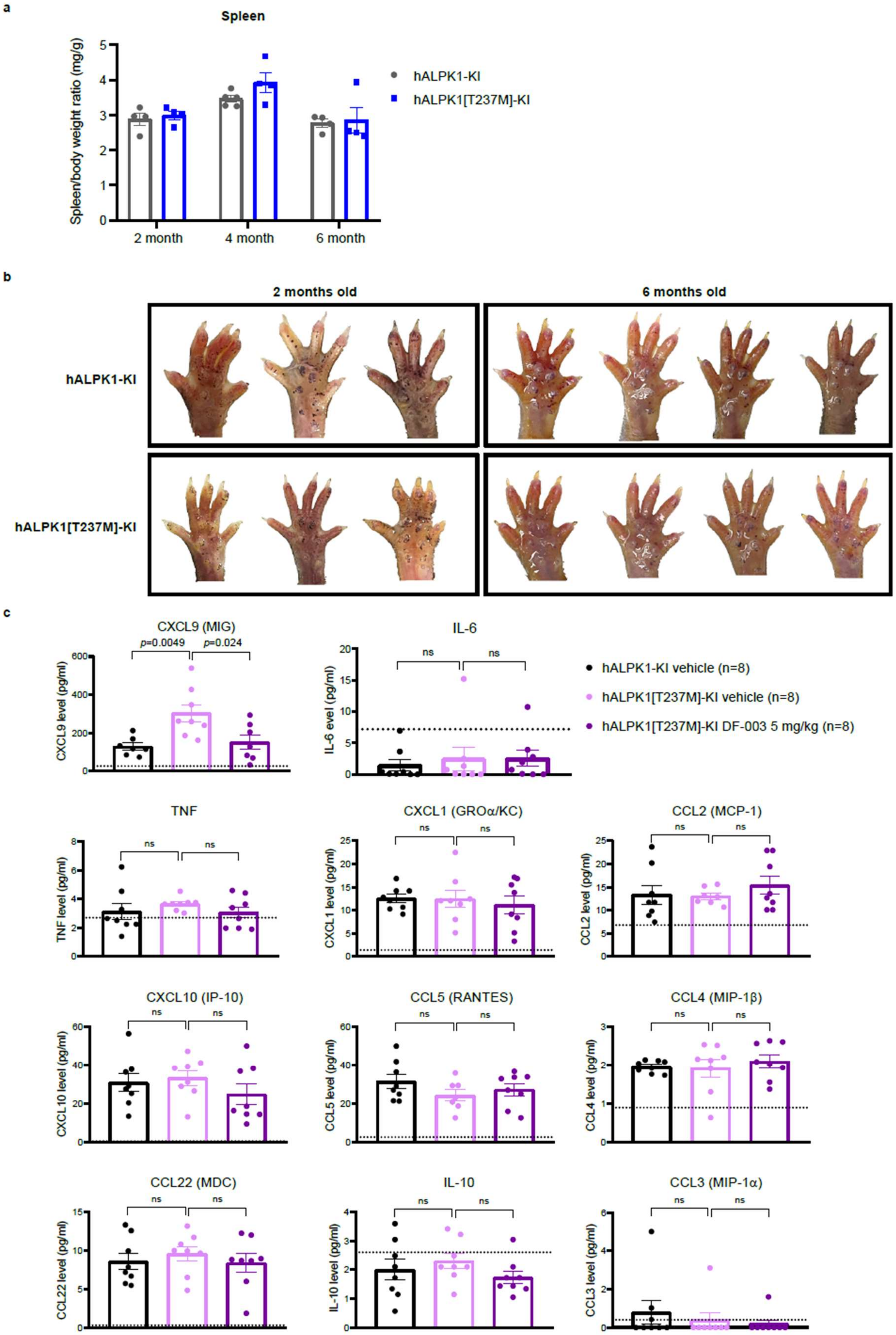
a
Spleen
Spleen/body weight ratio (mg/g)
2 month
4 month
6 month
hALPK1-KI
hALPK1[T237M]-KI
b
2 months old
6 months old
hALPK1-KI
hALPK1[T237M]-KI
c
CXCL9 (MIG)
p=0.0049
p=0.024
CXCL9 level (pg/ml)
IL-6
IL-6 level (pg/ml)
ns
hALPK1-KI vehicle (n=8)
hALPK1[T237M]-KI vehicle (n=8)
hALPK1[T237M]-KI DF-003 5 mg/kg (n=8)
TNF
TNF level (pg/ml)
CXCL1 (GROα/KC)
CXCL1 level (pg/ml)
CCL2 (MCP-1)
CCL2 level (pg/ml)
CXCL10 (IP-10)
CXCL10 level (pg/ml)
CCL5 (RANTES)
CCL5 level (pg/ml)
CCL4 (MIP-1β)
CCL4 level (pg/ml)
CCL22 (MDC)
CCL22 level (pg/ml)
IL-10
IL-10 level (pg/ml)
CCL3 (MIP-1α)
CCL3 level (pg/ml)

Supplementary Figure 5. ROSAH model mice do not exhibit splenomegaly, anhidrosis, or pronounced cytokine changes. a 2-month-, 4-month- and 6-month-old male hALPK1-KI or hALPK1[T237M]-KI mice, n=4 animals for each genotype at each age, were sacrificed, and spleens were resected and weighed. Spleen index values were measured as the ratio of spleen weight to body weight. **b** An iodine-starch sweat test revealed no obvious anhidrosis in hALPK1[T237M] mice foot pads, n=3 for each genotype at 2-month-old age and n=4 for each genotype at 6-month-old age. Only images of the right hind paw of each mouse were shown here. **c** Female hALPK1-KI and hALPK1[T237M]-KI mice were treated orally with vehicle or DF-003 5 mg/kg once per day for 10 days (n=8 animals for each group). Twenty-four hours after the last dose, the indicated plasma cytokine and chemokine levels were measured using a ProcartaPlex Kit. Data represent means ± SEM for each genotype and treatment group. The dotted lines represent the lower limit of quantification (lowest concentration of standards) for each cytokine/chemokine. Using Grubbs' test (α=0.05), for CXCL9, one animal in the hALPK1-KI group and one animal in the hALPK1[T237M]-KI DF-003 group were identified as outliers; for CCL2 and CCL5, one animal in the hALPK1[T237M] vehicle group was identified as an outlier. These outliers were removed from the statistical analyses. Statistical comparisons between groups were made with two-tailed Student's *t*-test: $^{*}p<0.05$; $^{**}p<0.01$; F=7.088, t=3.385, r^2=0.47, 95% CI 62.87 to 284.7, df=13, p=0.0049 between hALPK1-KI vehicle hALPK1[T237M]-KI groups; F=1.696, t=2.560, r^2=0.34, 95% CI -280.8 to -23.78, df=13, p=0.024 between hALPK1[T237M]-KI and hALPK1[T237M] 5 mg/kg groups.

Supplementary Table 1. Molecular Dynamics Simulations Checklist

**Reliability and reproducibility checklist for molecular dynamics simulations**<br>***All boxes must be marked YES by acceptance unless "Response not needed if No".**	**Yes**	**No**	**Response (Please state where this information can be found in the text)**
**1. Convergence of simulations and analysis**			
1a. Is an evaluation presented in the text to show that the property being measured has equilibrated in the simulations (*e.g.* time-course analysis)?	☒	☐	A time-course analysis of protein and ligand RMSD (root mean square deviation) was performed to monitor structural equilibration (See *Supplementary Note Fig. 4d*).
1b. Then, is it described in the text how simulations are split into equilibration and production runs and how much data were analyzed from production runs?	☒	☐	See *Supplementary Note Fig. 4d*)
1c. Are there at least 3 simulations per simulation condition with statistical analysis?	☒	☐	See *Main Text – Methods – Molecular*

			dynamics simulation of the ALPK1–DF-003 complex.
1d. Is evidence provided in the text that the simulation results presented are independent of initial configuration?	☐	☒	Two separate MD simulation workflows were performed. According to the experimental purpose, the stable conformation selection process used the initial homology modeling conformation, and the stability assessment process evaluated the selected complex conformation. In both workflows, the initial configuration was kept fixed.
**2. Connection to experiments**			
2a. Are calculations provided that can connect to experiments (*e.g.* loss or gain in function from mutagenesis, binding assays, NMR chemical shifts, J-couplings, SAXS curves, interaction distances or FRET distances, structure factors, diffusion coefficients, bulk modulus and other mechanical properties, *etc*.)?	☒	☐	See paragraph 6 of *Supplementary Note 1*. The high correlation between the docking scores and kinase inhibitory activities of the SAR study compounds demonstrates the accuracy of the complex conformations selected by MD.
**3. Method choice**			
3a. Do simulations contain membranes, membrane proteins, intrinsically disordered proteins, glycans, nucleic acids, polymers, or cryptic ligand binding?	☐	☒	Response not needed if **No**
3b. Is it described in the text whether the accuracy of the chosen model(s) is sufficient to address the question(s) under investigation (e.g. all-atom vs. coarse-grained models, fixed charge vs. polarizable force fields, implicit vs. explicit solvent or membrane, force field and water model, etc.)?	☒	☐	The model used in this paper adopts typical conditions for studying the interaction between compounds and protein complexes. The high correlation between the docking scores and kinase inhibitory activities of the SAR

			study compounds proved that the accuracy of the model is sufficient. See paragraph 6 of *Supplementary Note 1*.
3c. Is the timescale of the event(s) under investigation beyond the brute-force MD simulation timescale in this study that enhanced sampling methods are needed?	☐	☒	See *Supplementary Note Fig. 4d*). Within the scope of conventional MD simulations, the measured properties reach equilibrium very quickly in the simulation, so enhanced sampling methods are not required.
If **YES**, are the parameters and convergence criteria for the enhanced sampling method clearly stated?	☐	☐	
If **NO,** is the evidence provided in the text?	☒	☐	See *Supplementary Note Fig. 4d*). Within the scope of conventional MD simulations, the measured properties reach equilibrium very quickly in the simulation, so enhanced sampling methods are not required.
**4. Code and reproducibility**			
4a. Is a table provided describing the system setup that includes simulation box dimensions, total number of atoms, total number of water molecules, salt concentration, lipid composition (number of molecules and type)?	☒	☐	See *Supplementary Note Table. 8*
4b. Is it described in the text what simulation and analysis software and which versions are used?	☒	☐	See *Main Text – Methods – Molecular dynamics simulation of the ALPK1–DF-003 complex.*
4c. Are other parameters for the system setup described in the text, such as protonation state, type of structural restraints if applied, nonbonded cutoff, thermostat and barostat, etc.?	☒	☐	See *Main Text – Methods – Molecular dynamics simulation of the ALPK1–DF-003 complex.*
4d. Are initial coordinate and simulation input files and a coordinate file of the final output provided as supplementary files or in a public repository?	☒	☐	See *supplementary files*

4e. Is there custom code or custom force field parameters?		☐	☒	Response not needed if **No**
	If **YES**, are they provided as supplementary files or in a public repository?	☐	☐	

Supplementary Table 2. Pharmacokinetic parameters of DF-003 after 10th daily oral dosing in male C57BL/6J mice

	**3 mg/kg**	**5 mg/kg**
**C_{MAX} (ng/mL)**	160 ± 38.6	310 ± 73.5
**T_{MAX} (h)**	2.00 ± 0.00	2.67 ± 1.15
**$T_{1/2}$ (h)**	7.69 ± 0.99	8.81 ± 1.03
**CL/F (mL/min/kg)**	27.6 ± 1.94	21.6 ± 4.11
**$AUC_{0\sim TAU}$ (h*ng/mL)**	1815 ± 132.0	3944 ± 697.0
**VZ/F (L/kg)**	18.3 ± 1.55	16.5 ± 3.68
**C_{AVG} (ng/mL)**	75.6 ± 5.53	164 ± 29.0

Data are means ± SD (n = 3 mice per group).

Supplementary Table 3. qPCR primers used in this study.

Gene name	Sequence
**HUMAN**	
TNF	CTCTTCTGCCTGCTGCACTTTG
	ATGGGCTACAGGCTTGTCACTC
CXCL10	GGTGAGAAGAGATGTCTGAATCC
	GTCCATCCTTGGAAGCACTGCA
CXCL8	AAGGTGCAGTTTTGCCAAGG
	CCCAGTTTTCCTTGGGGTCC
GAPDH	AATTCCATGGCACCGTCAAG
	TGGACTCCACGACGTACTCA
**MOUSE**	
Ccl2	CCAATGAGTAGGCTGGAGAGC
	GACCCATTCCTTCTTGGGGTC
Ccl5	CTCACCATATGGCTCGGACA
	CGACTGCAAGATTGGAGCAC
Cxcl1	ACTCAAGAATGGTCGCGAGG
	GTGCCATCAGAGCAGTCTGT
Cxcl9	CCTAGTGATAAGGAATGCACGATG
	CTAGGCAGGTTTGATCTCCGTTC
Cxcl10	CCACGTGTTGAGATCATTGCC
	GAGGCTCTCTGCTGTCCATC
Cx3cr1	GTGAGTGACTGGCACTTCCT
	GACCGAACGTGAAGACGAGG
Aif1	TCTGCCGTCCAAACTTGAAGCC
	CTCTTCAGCTCTAGGTGGGTCT
Tnf	CCCTCACACTCAGATCATCTTCT
	GCTACGACGTGGGCTACAG
Rpl13	CTGCTCTCAAGGTTGTTCGGCT
	CCTTCCGTTTCTCCTCCAGAGT
mIl6	TACCACTTCACAAGTCGGAGGC
	CTGCAAGTGCATCATCGTTGTTC

Supplementary Note 1

Design of DF-003

To discover a suitable molecular starting point for the design of an ALPK1 kinase inhibitor, we developed a thermal shift assay (TSA) that measures ALPK1-ligand interactions. In a pilot TSA screen using a generic commercial kinase inhibitor library and an FDA-approved drug library (total of 1680 compounds; EFEBIO), AS-252424, among other hits (Supplementary Note Fig. 1a), was discovered to cause an increase in melting temperature (T_m) of 3.4°C. Surface plasmon resonance (SPR) analyses confirmed the reliability of using a TSA as a means of screening for ALPK1 binders. SPR and TSA results of representative hits, including AS-252424, sulfasalazine, TBB (tetrabromobenzotriazine), eltrombopag, novobiocin, niflumic acid, and tolcapone were summarized in Supplementary Note 1 Table 1. To survey diverse chemical structures for the ability to bind ALPK1, we screened a small molecule library of ~160,000 compounds (from WuXi AppTec) in a high-throughput TSA screen, leading to the identification of 243 compounds with binding activity (compounds with a TSA $T_m > 0.8$°C and drug-like structures). Next, these 243 hits were screened in a TIFA phosphorylation assay using Western blotting to measure the ability of compounds to inhibit the ALPK1-mediated phosphorylation of TIFA. This assay led to the identification of 18 compounds that inhibited ALPK1 kinase activity as potential hits (see Supplementary Note Fig. 1b for an example). We developed a TR-FRET (time-resolved Förster resonance energy transfer)-based ALPK1 kinase assay, in this TR-FRET assay Eltrombopag and Tolcapone showed IC_{50} of 7.6 and 20 μM, respectively. One of the 18 hit compounds, termed Hit 1, with an IC_{50} of 4 μM in the TR-FRET assay, emerged as a promising starting point for further investigation (Supplementary Note Fig. 1c).

Using Hit 1 as a starting point, a series of urea analogs (Supplementary Note Tables 2-4) was synthesized and evaluated. In general, the urea analogs of Hit 1 did not significantly impact potency (Supplementary Note Table 2). However, the results shown in Supplementary Note Tables 3-4 suggested that significant

molecular space was available for SAR development in the region of attachment of the benzylpiperazine moiety or alkyl alcohol moiety. Further SAR studies of the five-member ring between the phenyl and thiazole group of Hit 1 showed that when this moiety was replaced with smaller rings, such as a three-member ring (**7**) or four-member ring (**8**), a decrease in potency was observed. However, the five-member ring could tolerate being opened, and substitution of lipophilic groups was preferred. The SAR study on the opened five-member ring showed that when the R_2 group on the methylene position between the phenyl and thiazole group (R_2/R_3) was larger, this had a detrimental effect on potency (**70**). A decrease in potency was also noted with the R_2 substitution of H (**59**). This phenomenon indicated that the presence of a suitably sized lipophilic group at this methylene position is crucial for potency. Lipophilic substitutions such as methyl-ethyl or methyl-methyl combinations were associated with improved ALPK1 inhibitory activity. Other alkyl group combinations were also investigated, including ethyl-ethynyl, methyl-propynyl, methyl/methoxypropanyl, and methyl-ethynyl, the latter of which was associated with the best potency (Supplementary Note Tables 4, 7). Substitution on the phenyl ring of Hit 1 (Ring A) was additionally investigated. While the substitution of CN or OCF_3 groups resulted in decreased potency (Supplementary Note Table 3), halogen, CH_3, and OMe groups exhibited approximately equal activity levels (Supplementary Note Table 3). This SAR study indicated that the presence of an electron donating group was associated with more favorable activity than an electron withdrawing group. Compounds **5** and **6** clearly showed that *meta*-substitution negatively impacted inhibitor potency. SAR development around Hit 1 yielded the optimized compound **34** (IC_{50} = 48 nM) containing Pharmacophore 1 (Supplementary Note Table 4 and Supplementary Note Fig. 1c).

In a second high-throughput screen, a separate small molecule library of ~200,000 compounds (from WuXi AppTec) was screened using the TR-FRET ALPK1 kinase assay. Compound **23** (Supplementary Note Table 3) at 20 μM was used as a positive control in the screen. From this screen, 14 hits were identified. The IC_{50} values of these hits in the TR-FRET kinase were below 20 μM, and all hits exhibited drug-like

structures. Hit 2 (IC_{50} = 11 μM) was selected as being desirable for follow-up studies (Supplementary Note Fig. 1c). Hit 2 was further modified in an extensive SAR study (Supplementary Note Table 5) which revealed that the methoxy group at benzyl-thiazole position 7 adversely impacted inhibitory activity of the resultant compound. When this 7-methoxy substitution was removed (**35**), potency levels increased significantly (Supplementary Note Table 5). Subsequent SAR work focused on the para substitution (R_7) of the di-F phenyl ring of Hit 2. The results demonstrated that a basic group is crucial to improve potency. SAR optimization of Hit 2 led to the determination that piperazine substitution at the R_7 position of the di-F phenyl ring yielded the compound with the best activity (**43**), IC_{50} = 209 nM, containing Pharmacophore 2 (Supplementary Note Table 5 and Supplementary Note Fig. 1c). We conducted a further SAR study of Pharmacophore 1 by synthesizing a series of amide compounds (Supplementary Note Table 6). Finally, the combination of Pharmacophore 1 and Pharmacophore 2, as shown in Supplementary Note Fig. 1c and Supplementary Note Table 6, yielded DF-003 (**50**), which provided the most potent inhibition of ALPK1. Supplementary Note Fig. 1d shows the SPR sensorgram validating DF-003's binding to full length ALPK1, with a K_d of 90 nM (Supplementary Note 1 Table 1).

In order to conduct more in-depth SAR analyses and to further optimize DF-003, another array of compounds was synthesized around the Pharmacophore 1 portion of DF-003 (Supplementary Note Table 7). DF-003 was ultimately selected as a compound for further drug development based on its good inhibitory activity and other desirable development properties including its pharmacokinetics, drug metabolism, and toxicology profile.

To aid in the interpretation of SAR results we built a structure-based modeling application to understand the interactions of ALPK1 inhibitors with the binding pocket in this enzyme. A single crystal X-ray structure of the ALPK1 N-terminal domain (ALPK1-ND), ALPK1 kinase domain (mouse ALPK1-KD; mALPK1-KD), and the agonist ligand ADP-D-heptose was successfully generated. To date, the human

ALPK1-KD has not been amenable to crystal growth. While we attempted to co-crystalize ALPK1 inhibitors with the wild-type ALPK1 kinase domain, we were not successful.

Nonetheless, as mouse and human ALPK1-KD are highly conserved, sharing 89% amino acid sequence identity, we did successfully co-crystallize the human ALPK1-ND (residues 1-446) with the mALPK1-KD (residues 949-1231). The 2.25 Å structure of the complex was determined using selenomethionine-based single-wavelength anomalous dispersion (SAD) (Methods, *Crystallography*). The ALPK1-ND consists of 18 alpha helices (Supplementary Note Fig. 2a, b) and interacts with mAlpk1-KD in the co-crystal structure. Alpha-helices 14, 16, and 17 of the ALPK1-ND interact with the N-lobe of the mAlpk1-KD. The linking regions for α-helices 7, 8, 9, and 10 of the ALPK1-ND were found to interact with the C-lobe of mAlpk1-KD. ADP-D-heptose, originated from the *E. coli*. expressing the recombinant protein, was observed in a pocket of ALPK1-ND (Supplementary Note Fig. 2a, b). As described in the Methods, we developed a homology model of human ALPK1 kinase domain and conducted docking experiments to understand the interaction between DF-003 and the ALPK1 kinase domain. Much like the binding pocket established for an alpha-kinase reference protein, 3PDT, the structure of which was determined by single crystal X-ray structure analysis[1], the three consecutive lysine (LYS) residues (LYS1041, LYS1042, and LYS1043) in the p-loop structure of ALPK1 help form the unique molecular binding pocket of ALPK1 (Supplementary Note Fig. 2c). This unique attribute may explain the high kinase selectivity of our inhibitor DF-003. DF-003 was docked with a human ALPK1-KD homology model, and an analysis of the docking results revealed that DF-003 exhibited a high docking score (-8.493 kcal/mol), forming stable interactions with the key residues of the active site of ALPK1 (Supplementary Note Fig. 3a, b). The interactions formed by DF-003 included two hydrogen bonds (with GLY1136 and GLU1137), two π–π stacked interactions (with TYR1133 and PHE1138), and one salt bridge interaction with GLU1137 (Supplementary Note Fig. 3b). The hydrogen bond between amide NH and GLY1136 and π–π interactions ensure a hinge binding mode similar to that of ATP (Supplementary Note Fig. 3c and d), which contributes to the stability of the binding, while the piperazine salt bridge and hydrogen bond interactions further enhance its binding affinity and molecular

interactions (Supplementary Note Fig. 3d). Even though the interaction of the di-fluorine (di-F) substitution with an amino acid in the kinase domain pocket was not observed, as reported, the size of fluorine-containing functional groups is very unique[2] The size of fluorine groups can help direct a compound into its target pocket. Once the molecule is inside the binding pocket, the electronic characteristics of the fluorine group can then influence other binding moieties on the molecule to enhance the binding potency of the drug[2]. In our case, the di-F substitution might force the phenyl ring into a more suitable binding pose. When the compound lacks the di-F moiety, an obvious reduction in potency was noted (**45**). In addition, the electronic effect of the di-F group may affect the electron distribution of the piperazine ring, providing a better interaction with GLU1137 in the kinase pocket (Supplementary Note Fig. 4a). Although the interaction of the methyl-ethynyl group with the protein pocket was not observed in this molecular modeling effort, based on the results of the SAR study we concluded that ethynyl was a preferred lipophilic group conducive to a good compound fit in the deep kinase domain pocket (Supplementary Note Fig. 4b). Docking scores and kinase inhibition activity for the SAR study compounds that led to the development of DF-003 were strongly correlated (Supplementary Note Tables 2-7 and Supplementary Note Fig. 4c). The molecular dynamics of the ALPK1-DF-003 complex also revealed good stability, with a protein root mean square deviation (RMSD) < 3 Å (Supplementary Note Fig. 4d and Supplementary Note 1, Table 8).

To validate the molecular homology modeling, we tested whether the inhibitory potency of DF-003 would be altered using different mutant ALPK1 kinases. Site-directed mutagenesis was used to create single amino acid mutants of ALPK1 at positions predicted to mediate binding with DF-003 (Supplementary Note 1, Table 9). First, we tested whether expressing these mutant proteins in ALPK1 knock-out HEK293 cells could rescue ALPK1 agonist-induced NF-kB activation. Co-transfection of an NF-κB reporter construct and measurement of luciferase activity after addition of ALPK1 agonist DF-006 was used to assess NF-κB activation. GLU1137A, GLU1137K, and TYR1133A mutants retained at least partial ALPK1 activity, and we purified those mutant enzymes and measured the IC_{50} of DF-003 compared to wild-type ALPK1. DF-003 exhibited increased IC_{50} values of 44.6 nM, 23.7 nM, and 29.0 nM for GLU1137A, GLU1137K, and

TYR1133A, respectively, in contrast to an IC_{50} of 9.2 nM for the wild-type enzyme, suggesting reduced binding affinity of DF-003 to the three mutants (Supplementary Note 1, Table 9). These results are in line with the homology modeling prediction that GLU1137 and TYR1133 are involved in the binding of DF-003 to ALPK1.

Single amino acid substitution mutants at GLY1136 and PHE1138 failed to rescue the loss of NF-κB activation by DF-006 in ALPK1 knock-out cells, suggesting these mutants were kinase dead, likely because these residues are also critical for binding of ATP (Supplementary Note 1, Fig. 3). The result was not surprising as DF-003 is an ATP-competitive inhibitor and binds to the catalytic site of ALPK1. The residues predicted to interact with DF-003 in our computational model (TYR1133, GLY1136, GLU1137, and PHE1138) are either very close to catalytic amino acid residues or are catalytic residues themselves. Similarly, alpha kinase MHCK-A lost function when the conserved catalytic site PHE was mutated[1]. The single amino acid mutant of GLY1136 also led to loss of kinase activity, likely because it is also involved in hydrogen bonding between ALPK1 and ATP (Supplementary Note Fig. 3d). GLY1136 is also a conserved residue across all identified alpha kinases[2].

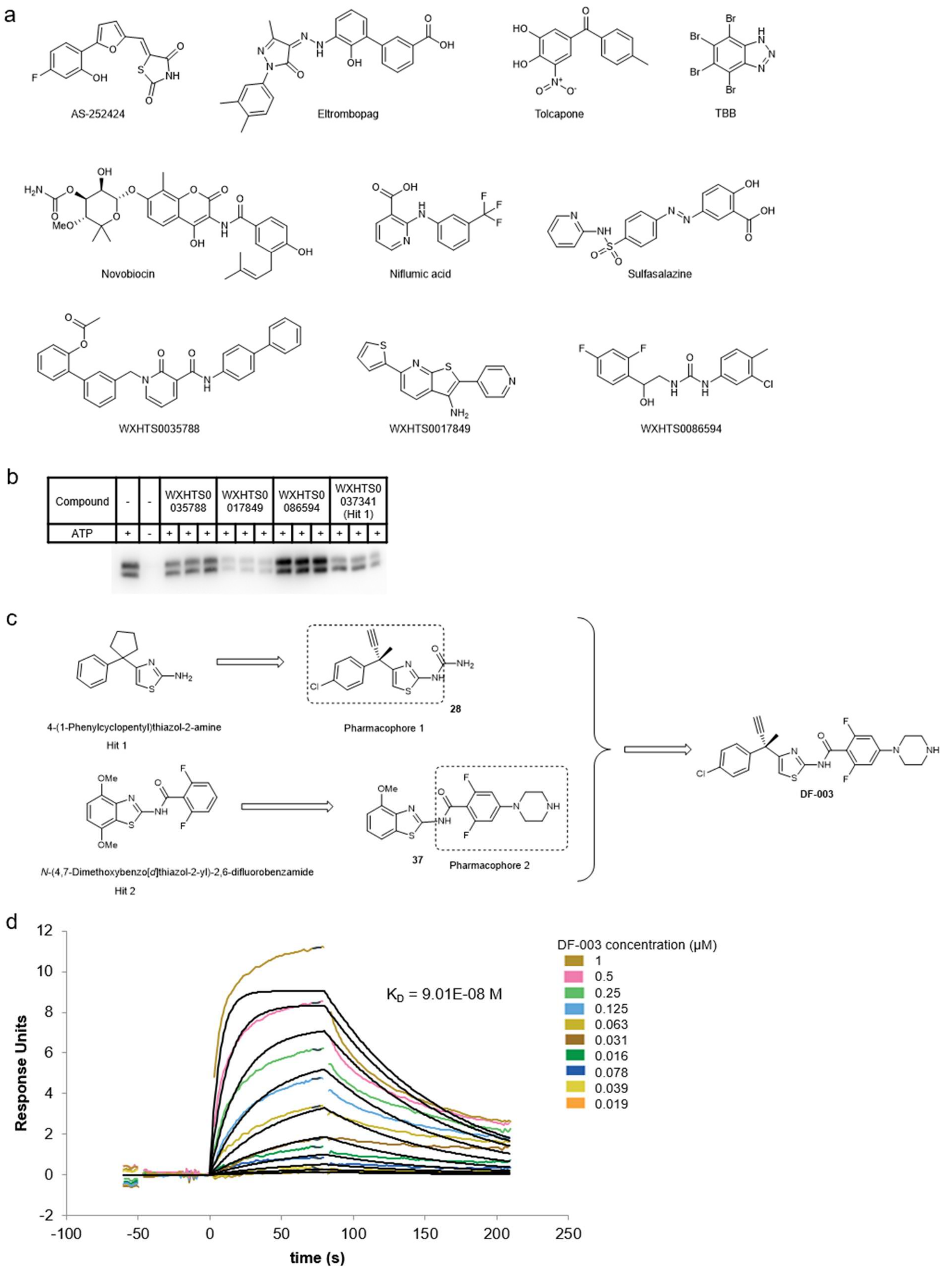

Supplementary Note Fig. 1. Design of DF-003 as an ALPK1 inhibitor.

(a) Structures of 7 pilot screening hits used in surface plasmon resonance (SPR) experiments and 3 compounds used in the Western blotting to measure the ability of compounds to inhibit the ALPK1-mediated phosphorylation of TIFA. (b) Example of the biochemical approach to validate hits from the thermal shift assay screen. ALPK1 binding compounds were tested in triplicate in an *in vitro* kinase assay at a final concentration of 20 μM, and the inhibitory effect was evaluated by comparing the phosphorylated TIFA level to that for the no compound control after the kinase reaction by Western blotting. Phosphorylated TIFA was detected using an anti-phospho-theronine antibody. ALPK1 binders fell into three categories: inhibitors (e.g. WXHTS0017849, WXHTS WXHTS0037341), activators (e.g. WXHTS0086594), and those with no effect (e.g. WXHTS0035788). (c) DF-003 was designed in a convergent manner starting with two independent high-throughput screens yielding Hits 1 and 2. An SAR approach improved ALPK1 inhibitory activity and led to two distinct Pharmacophores (1 and 2). DF-003 resulted from the combination of Pharmacophores 1 and 2. (d) Surface plasmon resonance (SPR) results for DF-003 with full-length ALPK1.

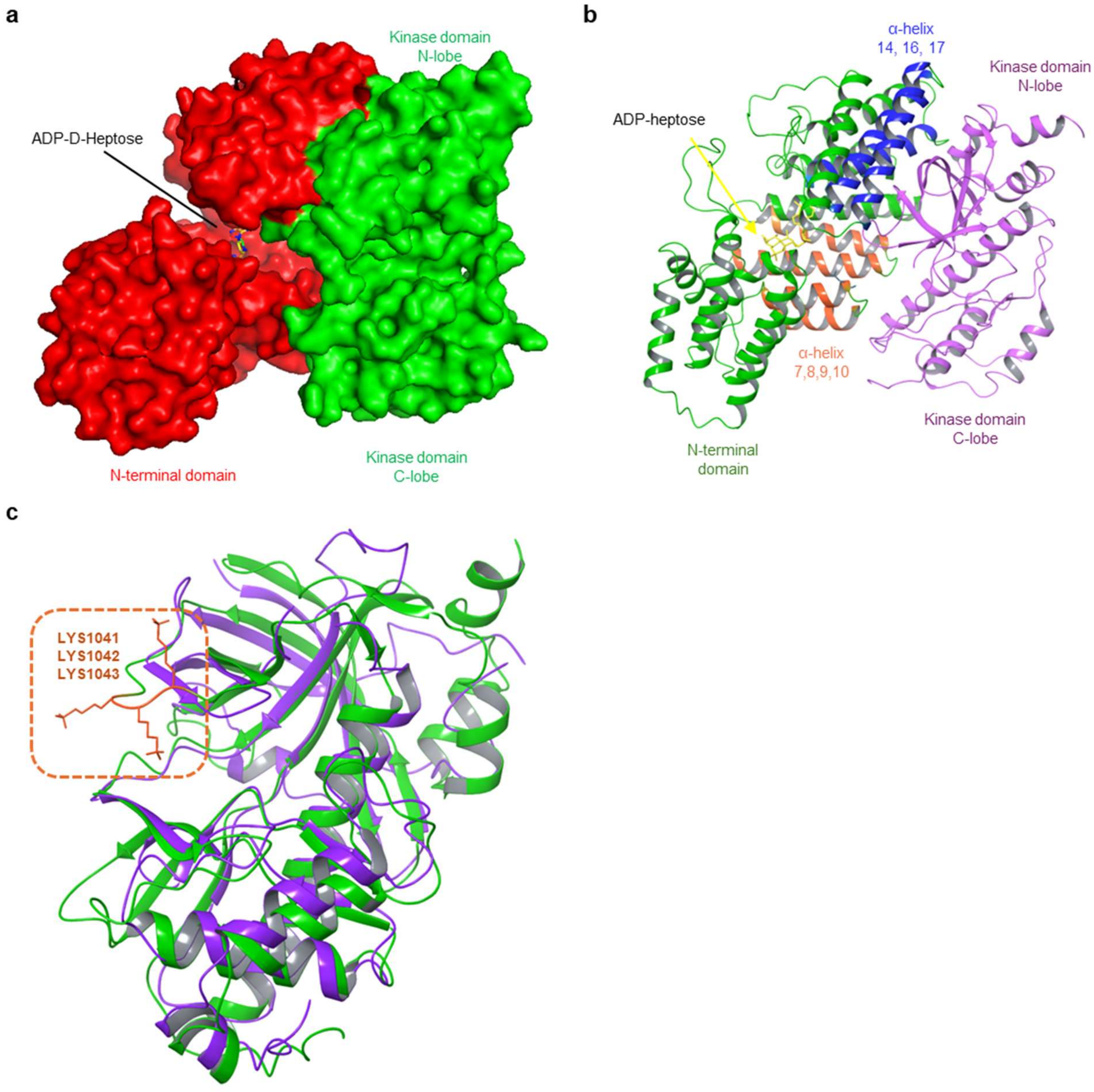

Supplementary Note Fig. 2. Structure of the ALPK1 complex and the kinase domain.

(a, b) Overall structure of the complex between hALPK1-N-terminal domain (ND) (1-446) and mALPK1-kinase domain (KD) (949-1230). hALPK1-ND (1-446) are shown in red (a) and rainbow (b), with mALPK1-KD in green (a) and magenta (b), and the ligand ADP-D-hptose is shown as a stick model (a). α-helices 7, 8, 9, and 10 of hALPK1-ND are shown in orange, and α-helices 14, 16, and 17 of hALPK1-ND are shown in blue. (c) Comparison of ALPK1 (human kinase domain) with 3PDT. The green ribbon

represents ALPK1, the purple ribbon represents 3PDT, the orange dotted box shows the unique three-LYS fragment in the ALPK1 p-loop.

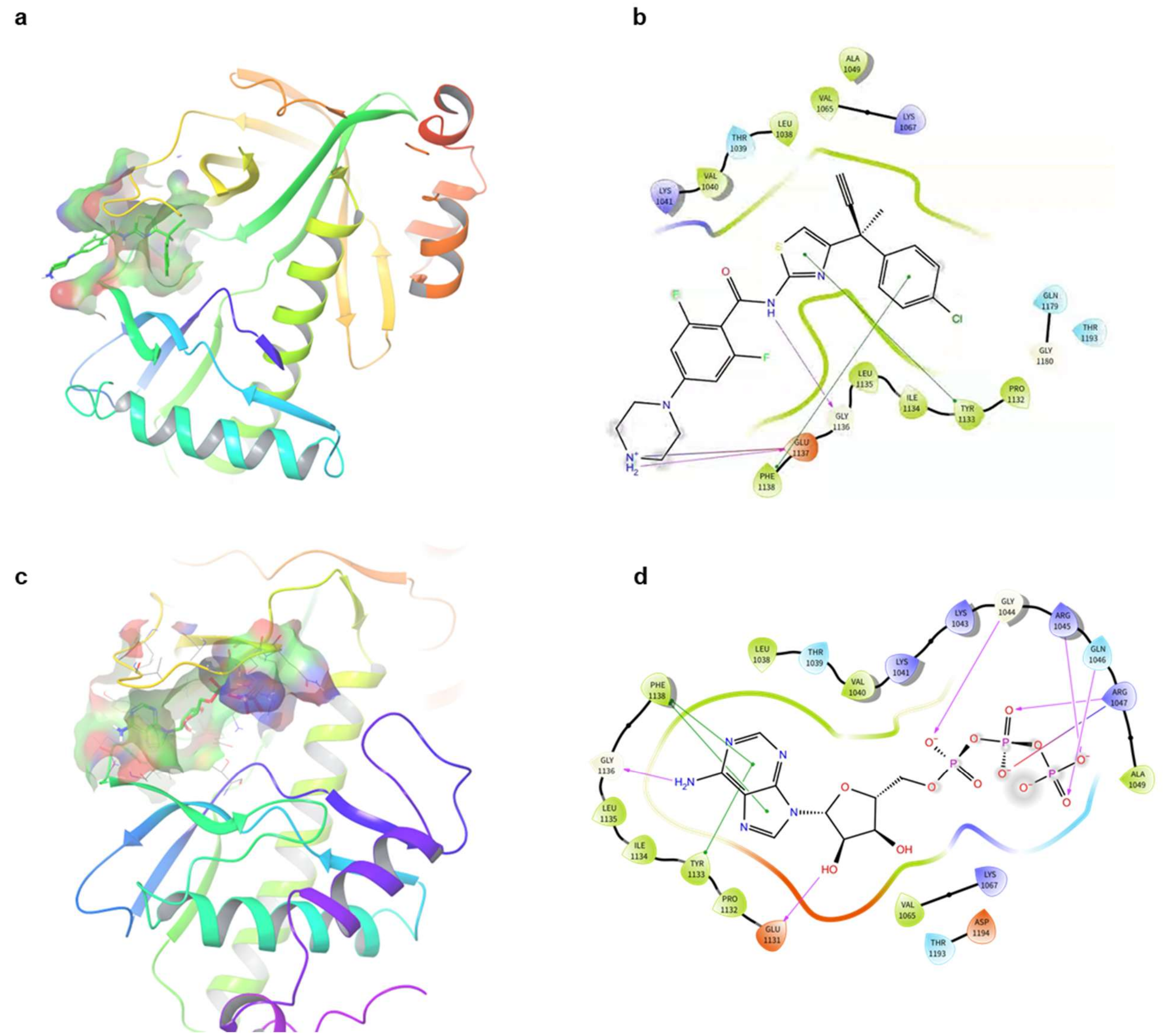

Supplementary Note Fig. 3. Structural modeling of interactions between DF-003 and ALPK1 and between ATP and ALPK1.

(a, b) Illustration of the interaction of DF-003 within the ALPK1 binding pocket: 3D view of the binding pose (a) and 2D ligand-interaction diagram (b). Green arrowed lines represent π–π stacking interactions; Purple arrowed lines represent hydrogen bond interactions; Blue arrowed lines represent salt bridge interactions. (c, d) A 3D view of the interaction of ATP within the ALPK1 binding pocket (c) and a 2D

ligand-interaction diagram (d). Green arrowed lines represent π–π stacking interactions; Magenta arrowed lines represent hydrogen bond interactions; Blue arrowed lines represent salt bridge interactions.

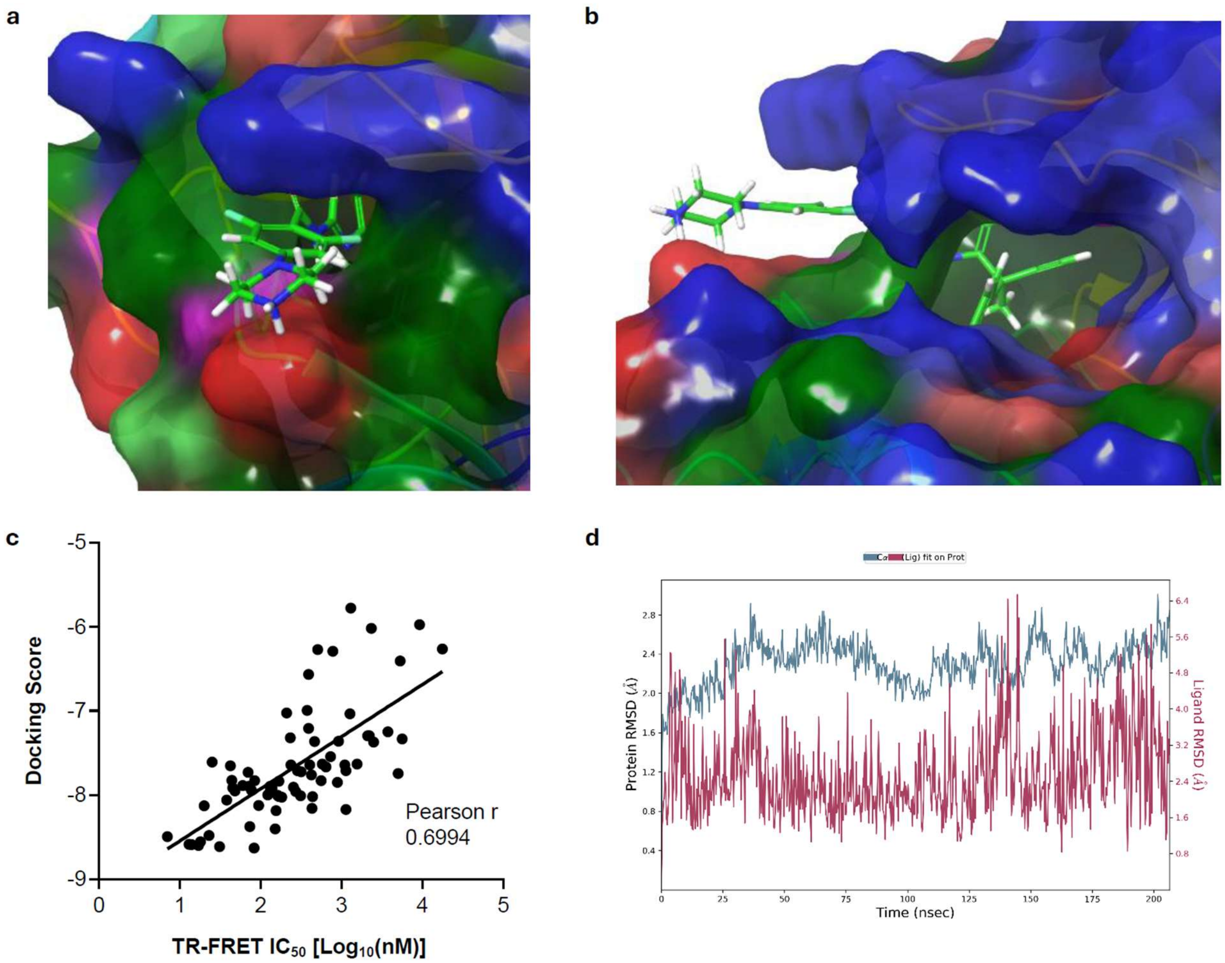

Supplementary Note Fig. 4. Molecular modeling of DF-003 within the ALPK1 binding pocket.

(a) In this example pose of DF-003, the di-F substitution helps place the phenylpiperazinyl moiety in a favorable position for a good interaction, especially of the piperazine ring, with the protein pocket. In addition, the di-F substitution provides an effect on the electron distribution of the piperazine ring, yielding a better interaction with GLU1137 in the kinase domain pocket (Supplementary Note Fig. 2d). (b) In this same example pose of DF-003, shown from a different perspective, the narrow and linearethynyl group provides a good fit into a deep kinase domain pocket. (c) Plots of docking scores and HTRF kinase IC_{50} results for ALPK1 inhibitors from Supplementary Note Tables 2-7. R^2 = 0.6994. (d) Time-course analysis of protein and ligand RMSD (root mean square deviation) of the ALPK1–DF-003 complex over a 200 ns molecular dynamics (MD) simulation. The first 100 ns were treated as the equilibration phase, while the subsequent 100 ns (100–200 ns) constituted the production phase. The protein root mean square deviation

(RMSD) during the production phase was less than 3 Å, and ligand RMSD aligned on the protein backbone was not significantly larger than the RMSD of the protein.

Supplementary Note Table 1. Binding affinity and thermal stability analysis of DF-003 and 7 pilot screening hits against full-length ALPK1.

Chemical name	SPR Kd (mM)	TSA T_m Increase (°C)
DF-003	0.00009	3.6
AS-252424	0.001	3.4
Sulfasalazine	0.281	2.0
Tbb	0.265	3.4
Eltrombopag	0.010	3.6
Novobiocin	0.010	1.4
Niflumic acid	0.007	1.8
Tolcapone	0.097	2.2

The table presents the dissociation constant (Kd) values in mM obtained from SPR measurements. TSA results are shown as the increase in melting temperature (T_m) inn°C for each compound at 10 μM.

Supplementary Note Table 2: SAR around ring A, R_2/R_3, and R_4 of benzyl urea

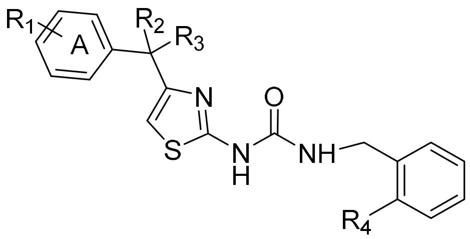

Compd ID	R_1	R_2/R_3	R_4	HTRF Assay (nM)	Docking Score
1	No substitution	cyclopentane	H	5000	7.746
2	No substitution	CH_3/CH_3	H	2500	7.371
3	No substitution	CH_3/CH_3	H	2100	-7.298
4	No substitution	CH_3/CH_3	OMe	2200	-7.298
5	*p*-Br	CH_3/CH_3	OMe	1100	-7.643
6	*m*-Br	CH_3/CH_3	OMe	No activity	-6.936
7	No substitution	Cyclopropane	OMe	No activity	-7.356
8	No substitution	Cyclobutane	OMe	No activity	-7.664
9	No substitution	CH_3/H	OMe	3750	-7.251

Supplementary Note Table 3: SAR around ring A and R_2/R_3 of piperazinyl benzyl urea

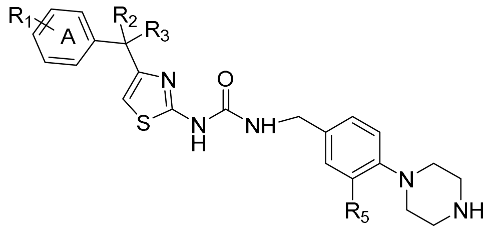

Compd ID	R_1	R_2/R_3	R_5	HTRF Assay (nM))	Docking Score
10	*p*-OCF_3	CH_3/CH_3	H	1132	-8.172
11	*p*-Cl	CH_3/CH_3	H	44	-7.825
12	*p*- Cyclopropane	CH_3/CH_3	H	155	-8.187
13	*p*-CN	CH_3/CH_3	H	236	-7.642
14	*p*-F	CH_3/CH_3	H	915	-7.363
15	*p*-I	CH_3/CH_3	H	284	-7.71
16	*p*-Br	CH_2OH/H	H	5620	-7.335
17	*p*-Br	CH_3/H	H	391	-6.569
18	*p*-Br	CH_3/CH_3	H	130	-7.962
19	*p*-Br	CH_3/C_2CH_5	H	429	-8.159
20	*p*-OMe	CH_3/CH_3	H	122	-7.999
21	*p*-OMe	CH_3/CH_3	F	84	-7.831
22	*P*-OH	CH_3/CH_3	H	732	-7.545
23	*p*-Br	CH_3/CH_3	F	138	-7.917
24	*p*-Br	CH_3/CH_3	Cl	233	-7.321
25	*p*-Br	CH_3/CH_3	CH_3	462	-7.366
26	*p*-Br	CH_3/CH_3	OMe	161	-8.004

Supplementary Note Table 4: SAR around ring A and R_2/R_3 of alkyl urea

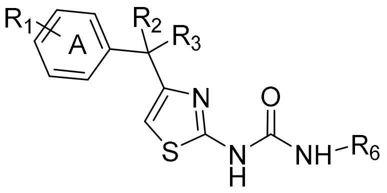

Compd ID	R_1	R_2/R_3	R_6	HTRF Assay (nM)	Docking Score
27	*p*-Br	CH_3/CH_3	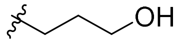	168	-7.838
28	*p*-Br	CH_3/CH_3	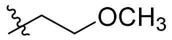	1130	-7.711
29	*p*-Br	Methyl/Ethynyl	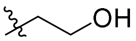	71	-7.898
30	*p*-Br	Methyl/Ethynyl	H	130	-7.897
31	*p*-OMe	Methyl/Ethynyl	H	94	-8.126
32	*p*-Br	Ethyl/Ethynyl	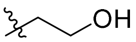	886	-7.85
33	*p*-Cl	Methyl/Methoxy-methyl	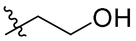	558	-7.83
34	*p*-Cl	Methyl/Ethynyl	H	48	-7.952

Supplementary Note Table 5: SAR study around the para-substitution of di-F phenyl ring part of Hit 2

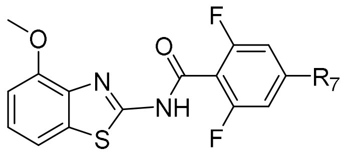

Compd ID	R_7	HTRF Assay (nM)	DOCKING SCORE
35	H	1300	-5.777
36	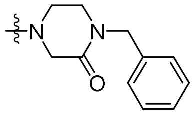	321	-6.297
37	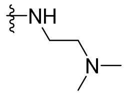	507	-6.274
38	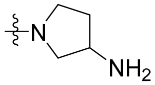	125	-7.211
39	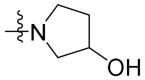	3190	-6.269
40	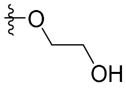	560	-6.017
41	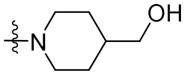	2278	-5.974
42	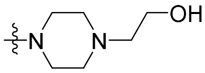	5306	-6.409
43	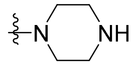	209	-7.027
44	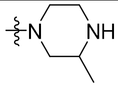	349	-6.998

Supplementary Note Table 6: SAR around Pharmacophore 1

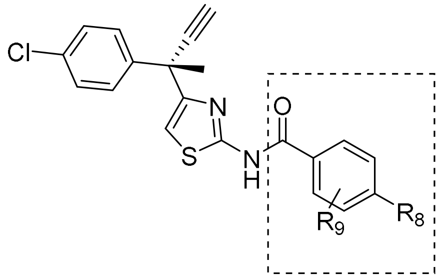

Compd ID	Fragment(R_8/R_9)	HTRF Assay (nM)	DOCKING SCORE
45	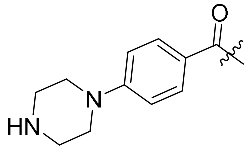	38	-8.059
46	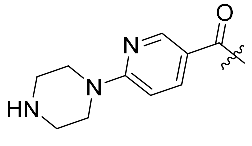	167	-8.015
47	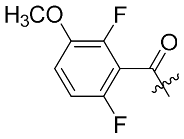	1549	-7.635
48	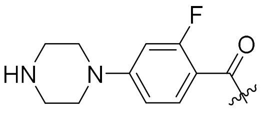	151	-8.403
49	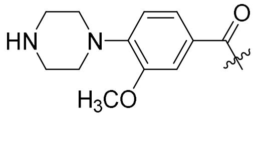	253	-7.907
50 (DF-003)	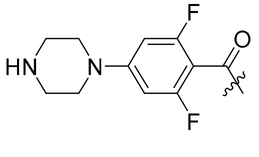	7	-8.493

51	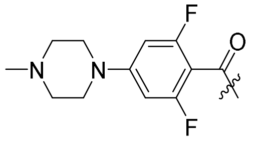	425	-7.761
52	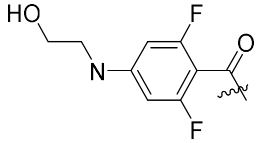	405	-7.643
53	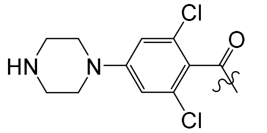	312	-8.01
54	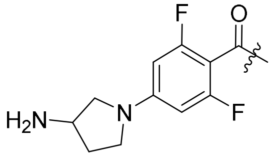	31	-8.611
55	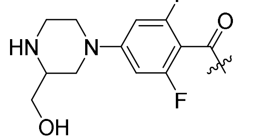	317	-7.725
56	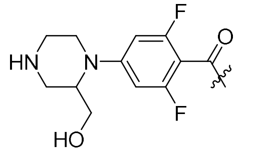	20	-8.129
57	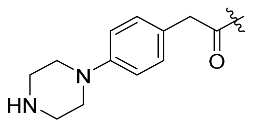	60	-7.889
58	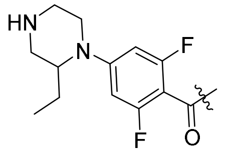	70	-7.73

Supplementary Note Table 7: SAR of the Pharmacophore 1 Portion of DF-003

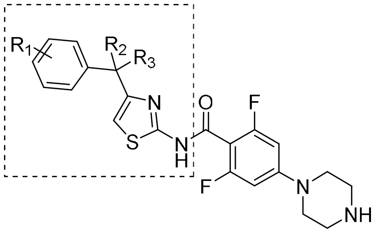

Compd ID	Fragment	HTRF Assay (nM)	DOCKING SCORE
59	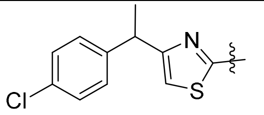	270	-7.959
60	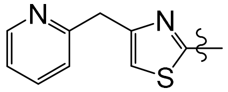	1271	-7.037
61	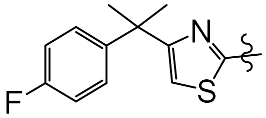	23	-8.48
62	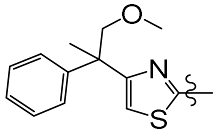	182	-8.027
63	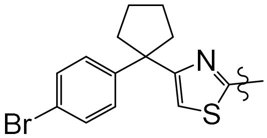	437	-8.019
64	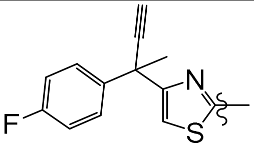	14	-8.589
65	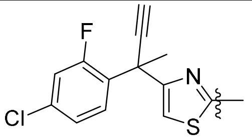	77	-7.945
66	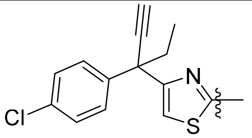	83	-8.63

67	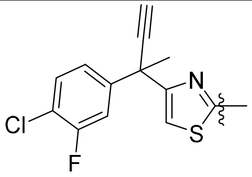	17	-8.6
68	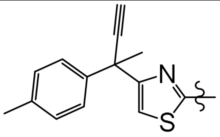	13	-8.586
69	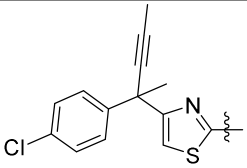	45	-7.914
70	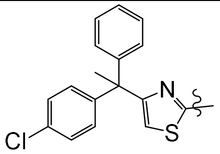	577	-7.633
71	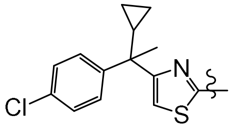	25	-7.609
72	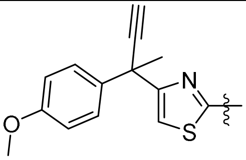	18	-8.554
73	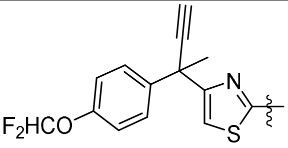	73	-8.375
74	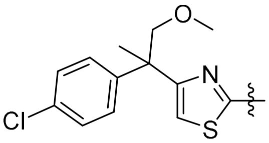	42	-7.655
75	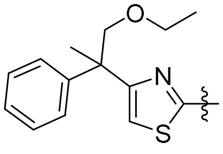	642	-7.667

Supplementary Note Table 8: Molecular dynamics simulation system setup of ALPK1-DF-003 complex

Parameter	Value / Description
Initial structure	ALPK1-DF-003 complex
Software	Schrodinger Release 2021-02, Desmond module
Simulation box type	Orthorhombic box generated with 10 Å padding in each direction
Simulation box volume	Minimized volume
Solvent model	SPC
Salt concentration	0.15 M NaCl
Neutralization	Na^+/Cl^- ions added to neutralize total system charge
Total number of atoms	33851
Total number of water molecules	9743
Force field	OPLS4
Simulation time	200 ns
Recording interval	200 ps
Ensemble class	NPT
Temperature	300 K
Pressure	1.01325 bar
Lipid composition	Not applicable

Supplementary Note Table 9: IC_{50}s of DF-003 on ALPK1 mutants.

	Change to	NF-κB activation after the addition of DF-006 when expressed in ALPK1 knock-out cells (% of cells expressing WT)	IC_{50} of DF-003 in kinase assay (nM)
WT		100%	9.2
TYR1133	A	19%	29.0
	Q	0	Kinase not purified
PHE1138	A	0	Kinase not purified
	Q	0	Kinase not purified
GLU1137	A	36%	44.6
	K	49%	23.7
GLY1136	A	0	Kinase not purified

Supplementary Note 2

Chemical Synthesis, Characterization, NMR Spectra, and Crystal Structure of DF-003

All reagents and solvents were used without further purification. NMR spectra were collected on a Bruker 400 MHz, or a Bruker 600 MHz spectrometer at ambient temperature; chemical shifts (δ) are reported in ppm downfield from tertramethylsilane, using the solvent resonance as the internal standard. Multiplicities are reported with the following abbreviations: s singlet; d doublet; t triplet; m multiplet; br broad; dd doublet of doublets.

Overview of DF-003 Synthesis

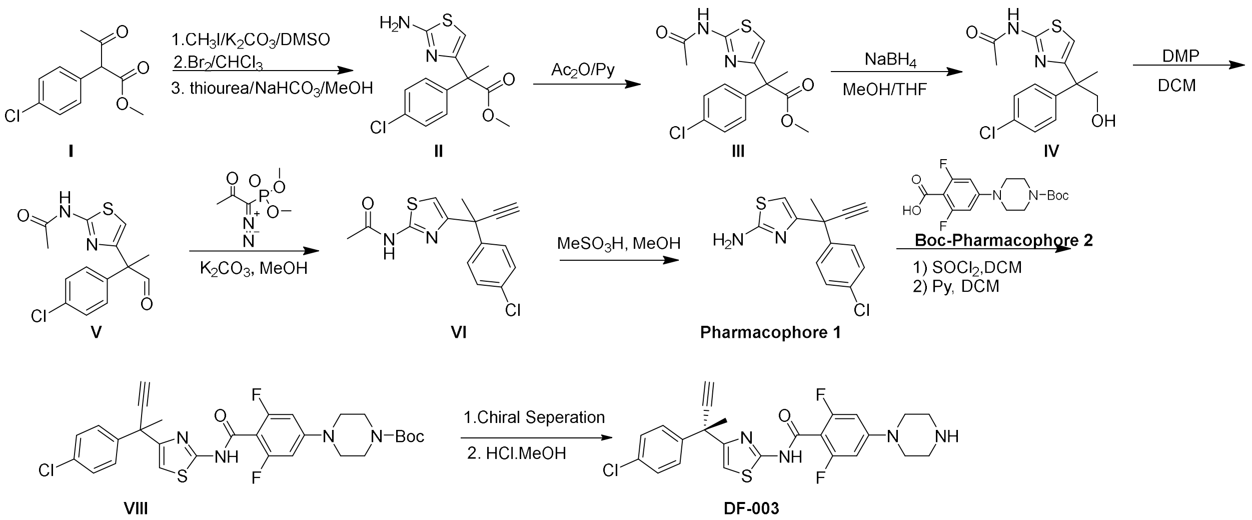

Procedure for the synthesis of methyl 2-(2-aminothiazol-4-yl)-2-(4-chlorophenyl)propanoate (II)

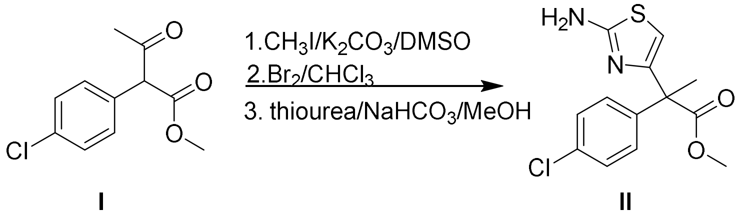

To a solution of methyl 2-(4-chlorophenyl)-3-oxobutanoate (compound **I**) (67 g, 0.296 mol) and K_2CO_3 (49.05 g, 0.356 mol) in dry DMSO (335 mL), iodomethane (50.5 g, 0.356 mmol, 22.1 mL) was slowly added at 15-25°C under N_2. The mixture was stirred at 25°C for 2-4 h. Subsequently, the mixture was

poured into ice-water (1.34 L) and ethyl acetate (EA; 1 L). After stirring for 30 min, two layers were separated, the organic phase was washed with H_2O (1 L), brine (1 L), dried over anhydrous Na_2SO_4, filtered, and concentrated. The crude product was dissolved in $CHCl_3$ (575 mL), then Br_2 (42.5 g, 0.27 mol, 14.6 mL) was slowly added under N_2. The mixture was stirred at 75°C for 3 h. TLC showed the reaction was completed. Saturated $NaHCO_3$ (350 mL) was added at 0-10°C, and the solution was extracted with DCM (210 mL), washed with H_2O (280 mL), dried over anhydrous Na_2SO_4, filtered, and concentrated to yield an orange oil. This orange oil was dissolved in MeOH (546 mL) and thiourea (30.45 g, 0.4 mol) was added, followed by the addition of $NaHCO_3$ (33.65 g, 0.4 mol) under N_2. The mixture was stirred at 50°C for 4 h. After filtering, the filtrate was concentrated to remove the volatiles. The remaining solid was triturated in H_2O (800 mL) and collected by filtration. Crude solids were dissolved in dichloromethane (DCM; 700 mL), washed with H_2O (200 mL) dried over anhydrous Na_2SO_4, and filtered. Active carbon (9.5 g) was added to the filtrate and stirred at room temperature for 2 h. After filtering, the filtrate was concentrated to obtain wet orange solids (78 g). The resulting material was then slurried in heptane (400 mL) and MTBE (4 mL) for 2 h. Filtration yielded a yellow solid (54 g). The crude product was recrystallized in ACN (108 mL). Compound **2** (36 g, purity 97%, yield for 4 steps: 40.7%) was obtained as an off-white solid. The purity was confirmed by HPLC with the following conditions: Agilent 1260 HPLC with UV detector and a detection wavelength of 220 nm; Column: Bridge C18 (150 mm * 4.6 mm, 3.5 μm) PN: 186003034; Column Temperature: 35ºC; Mobile Phase A:10 mM NH_4OAc in Water; Mobile Phase B: ACN (20%-95%). **^{1}H NMR** (400 MHz, $CDCl_3$) δ 7.30-7.27 (m, 2H), 7.25-7.21 (m, 2H), 6.13 (s, 1H), 5.21 (br s, 2H), 3.73 (s, 3H), 1.89 (s, 3H). **^{13}C NMR** (101 MHz, $CDCl_3$) δ 174.12, 167.25, 153.55, 140.79, 133.05, 128.91, 128.29, 105.31, 54.23, 52.69, 25.09. **HRMS** (ESI) m/z calcd. for $C_{13}H_{14}ClN_2O_2S^+$ $[M+H]^+$: 297.0459, found 297.0397.

Procedure for the synthesis of methyl 2-(2-acetamidothiazol-4-yl)-2-(4-chlorophenyl)propanoate (III)

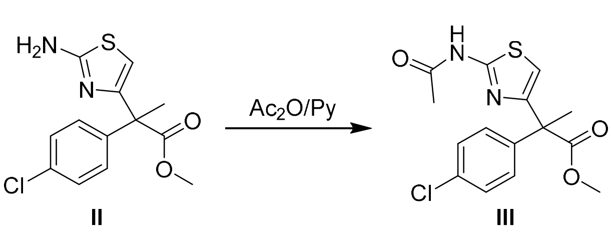

Methyl 2-(2-aminothiazol-4-yl)-2-(4-chlorophenyl)propanoate (compound **II**) (36 g, 0.122 mol) was dissolved in dry pyridine (288 mL), followed by the addition of acetic anhydride (49.6 g, 0.486 mmol, 46 mL) and DMAP (15 mg, 0.12 mmol) at 0=5 °C under N_2. The mixture was stirred at 25°C for 18 h. After pouring into ice-water (2.9 L), a yellow oil formed, and the aqueous phase was separated. The oil was

dissolved in EA (600 mL), washed with 0.5 N HCl (300 mL), saturated $NaHCO_3$ (300 mL), brine (300 mL), dried over anhydrous Na_2SO_4, filtered, and concentrated to give a yellow solid (45 g). The crude product was triturated in heptane (360 mL) and MTBE (3.6 mL) to generate compound **III** (36.1 g, purity 95%, yield: 87.8%) as an off-white solid. The purity was confirmed by HPLC with the following conditions: Agilent 1260 HPLC with a UV detector and a detection wavelength of 220 nm; Column: Bridge C18 (150 mm * 4.6 mm, 3.5 µm) PN: 186003034; Column Temperature: 35°C; Mobile Phase A:10 mM NH_4OAc in Water; Mobile Phase B: ACN (20%-95%). **^{1}H NMR** (400MHz, $CDCl_3$).δ 9.13 (br s, 1H), 7.30-7.26 (m, 2H), 7.20-7.17 (m, 2H), 6.60 (s, 1H), 3.71 (d, J = 1.1 Hz, 3H), 2.19 (s, 3H), 1.92 (s, 3H). **^{13}C NMR** (101 MHz, $CDCl_3$) δ 174.23, 167.74, 157.19, 152.66, 140.83, 133.15, 128.85, 128.35, 110.40, 54.17, 52.75, 25.24, 23.11. **HRMS** (ESI) m/z calcd. for $C_{15}H_{15}ClN_2O_3SNa^+$ $[M+Na]^+$: 361.0384, found 361.0340.

Procedure for the synthesis of N-(4-(2-(4-chlorophenyl)-1-hydroxypropan-2-yl)thiazol-2-yl)acetamide (IV)

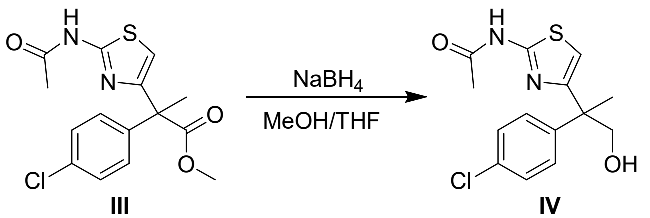

To a solution of methyl 2-(2-acetamidothiazol-4-yl)-2-(4-chlorophenyl)propanoate (compound **III**) (23 g, 68.04 mmol) in dry THF (230 mL), $NaBH_4$ (38.81 g, 1.02 mol) was added. The mixture was heated to 45°C under N_2. MeOH (2.75 mL, 1 eq.) was added and the reaction proceeded for 3 days. After cooling down, EA (150 mL) was added, followed by the dropwise addition of aq. 6 N HCl (145 mL) until reaching pH 7-8 at 5-20°C. Under these conditions, a white solid precipitated from the solution and gas was generated. H_2O (100 mL) was added, and the organic phase was separated. The aqueous phase was extracted with EA (200 mL). The combined organic phases were washed with brine (150 mL), dried over anhydrous Na_2SO_4, and concentrated to yield a crude yellow oil. The residue was purified by flash silica gel chromatography (PE: EA = 1: 0 to 2: 3). Compound **IV** (15.4 g, yield: 72.8%) was obtained as a pale yellow solid. **^{1}H NMR** (400 MHz, $CDCl_3$) δ 11.01 (br s, 1H), 7.23-7.18 (m, 2H), 7.11-7.08 (m, 2H), 6.55 (s, 1H), 4.13 (dd, J = 11.1, 1.3 Hz, 1H), 3.85 (d, J = 11.0 Hz, 1H), 2.15 (s, 3H), 1.60 (s, 3H). **^{13}C NMR** (101 MHz, $CDCl_3$) δ 168.22, 158.48, 155.61, 143.17, 132.51, 128.45, 128.27, 108.77, 71.53, 46.25, 24.69, 22.96. **HRMS** (ESI) m/z calcd. for $C_{14}H_{15}ClN_2O_2SNa^+$ $[M+Na]^+$: 333.0435, found 333.0429.

Procedure for the synthesis of N-(4-(2-(4-chlorophenyl)-1-oxopropan-2-yl)thiazol-2-yl)acetamide (V)

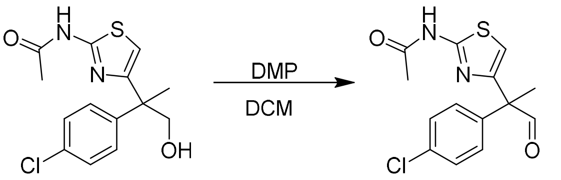

N-(4-(2-(4-chlorophenyl)-1-hydroxypropan-2-yl)thiazol-2-yl)acetamide (compound **IV**) (27 g, 86.8 mmol) was dissolved in DCM (400 mL). The mixture was chilled to 0°C, DMP (43.1 g, 101.6 mmol) was added in portions within 0.5 h. The mixture was then stirred at 25°C for 3 h, chilled to 5°C again, and saturated $Na_2S_2O_3$ (200 mL)/saturated $NaHCO_3$ (150 mL) was added. After extracting with DCM (200 mL), the combined DCM layers were washed with saturated $Na_2S_2O_3$ (100 mL)/saturated $NaHCO_3$ (100 mL), brine (150 mL), dried over anhydrous Na_2SO_4 and concentrated to give compound **V** as a yellow oil (23.4 g, yield: 87%). **^{1}H NMR** (400 MHz, $CDCl_3$) δ 9.97 (s, 1H), 9.63 (br s, 1H), 7.29-7.26 (m, 2H), 7.03-7.00 (m, 2H), 6.76 (d, J = 1.4 Hz, 1H), 2.25 (s, 3H), 1.73 (s, 3H). **^{13}C NMR** (101 MHz, $CDCl_3$) δ 200.07, 168.18, 158.06, 150.68, 139.20, 133.50, 128.92, 111.01, 57.37, 23.11, 21.44. **HRMS** (ESI) m/z calcd. for $C_{14}H_{13}ClN_2O_2SNa^+$ $[M+Na]^+$: 331.0278, found 331.0272.

Procedure for the synthesis of N-(4-(2-(4-chlorophenyl)but-3-yn-2-yl)thiazol-2-yl)acetamide (VI)

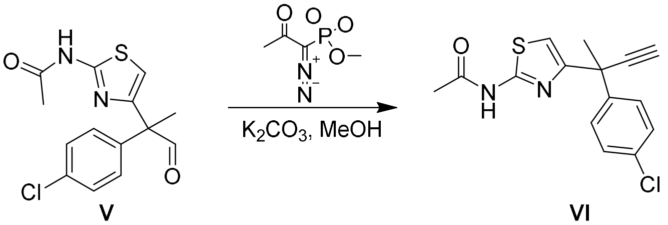

To a solution of N-(4-(2-(4-chlorophenyl)-1-oxopropan-2-yl)thiazol-2-yl)acetamide (compound **V**) (23.2 g, 75.31 mmol) in MeOH (350 mL), dimethyl (1-diazo-2-oxopropyl)phosphonate (21.7 g, 112.97 mmol) and K_2CO_3 (20.8 g, 150.63 mmol) were added at 0°C. The mixture was stirred at 25°C for 16 h, filtered, and concentrated to produce a brown oil. EA (150 mL) and H_2O (150 mL) were added, the organic phase was separated, and the aqueous phase was extracted with EA (100 mL). The combined organic layers were washed with 0.05N HCl (100 mL), sat. $NaHCO_3$ (100 ml), brine (100 mL), dried over anhydrous Na_2SO_4, and concentrated to yield a sticky solid (24 g). The crude product was purified by flash column chromatography (EA:PE=0-30%) to yield compound **VI** (16.1 g, purity 97.2%, yield 70.4%) as a yellow solid. The purity was confirmed by HPLC with the following conditions: Agilent 1260 HPLC with a UV

detector and a detection wavelength of 220 nm; Column: Bridge C18 (150 mm * 4.6 mm, 3.5 μm) PN: 186003034; Column Temperature: 35ºC; Mobile Phase A:10 mM NH4OAc in Water; Mobile Phase B: ACN (20%-95%). **^{1}H NMR** (400 MHz, $CDCl_3$) δ 9.06 (br s, 1H), 7.41-7.38 (m, 2H), 7.27-7.23 (m, 2H), 6.89 (s, 1H), 2.56 (s, 1H), 2.18 (s, 3H), 1.93 (s, 3H). **^{13}C NMR** (101 MHz, $CDCl_3$) δ 167.62, 157.75, 153.88, 142.58, 132.83, 128.38, 128.00, 109.09, 87.51, 72.50, 42.53, 29.47, 23.15. **HRMS** (ESI) m/z calcd. for $C_{15}H_{13}ClN_2OSNa^+$ $[M+Na]^+$: 327.0329, found 327.0322.

Procedure for the synthesis of 4-(2-(4-chlorophenyl)but-3-yn-2-yl)thiazol-2-amine (Pharmacophore 1)

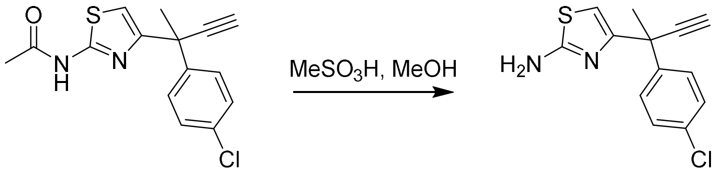

To a solution of N-(4-(2-(4-chlorophenyl)but-3-yn-2-yl)thiazol-2-yl)acetamide (compound **VI**) (16 g, 52.62 mmol) in MeOH (190 mL), $MeSO_3H$ (17.1 mL, 0.26 mol) was added at 25°C. The mixture was stirred at 60°C for 16 h. After cooling down to 0°C, $NaHCO_3$ (22.3 g) was added incrementally until the reaction solution pH reached 7. The reaction was concentrated to remove MeOH, EA (100 mL) and H_2O (100 mL) were added, phases were separated, and the aqueous phase was extracted with EA (100 mL). The combined organic layers were washed with brine (100 mL), dried over anhydrous Na_2SO_4, concentrated, and dried under vacuum to produce a residue. This residue was purified by flash silica gel chromatography (PE: EA = 1: 0 to 2: 1). **Pharmacophore 1** (13.4 g, yield 96.8%, purity 95.2%) was obtained as a pale yellow solid. The purity was confirmed by HPLC with the below conditions: Agilent 1260 HPLC with a UV detector and a detection wavelength of 220 nm; Column: Bridge C18 (150 mm * 4.6 mm, 3.5 μm) PN: 186003034; Column Temperature: 35ºC; Mobile Phase A:10 mM NH4OAc in Water; Mobile Phase B: ACN (20%-95%). **^{1}H NMR** (400 MHz, Chloroform-*d*) δ 7.42 -7.38 (m, 2H), 7.27- 7.24 (m, 2H), 6.36 (s, 1H), 5.47 (br s, 2H), 2.52 (s, 1H), 1.87 (s, 3H). **^{13}C NMR** (101 MHz, $CDCl_3$) δ 168.42, 154.74, 142.71, 132.71, 128.35, 128.09, 103.50, 87.74, 72.32, 42.57, 29.34. **HRMS** (ESI) m/z calcd. for $C_{13}H_{12}ClN_2S^+$ $[M+H]^+$: 263.0404, found 263.0348.

Procedure for the synthesis of 4-(4-(tert-butoxycarbonyl)piperazin-1-yl)-2,6-difluorobenzoic acid (Pharmacophore 2)

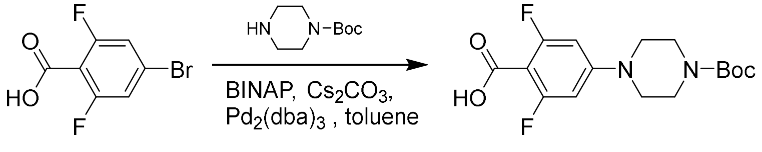

4-Bromo-2,6-difluorobenzoic acid (compound **VII**, 50 g, 0.21 mol), tert-butyl piperazine-1-carboxylate (45 g, 0.24 mol), Cs_2CO_3 (17.5 g), $Pd(OAc)_2$ (90 mg), and BINAP (0.39 g) were mixed in 1, 4-dioxane (900 mL). The mixture was stirred at 85-95°C for 16 h, then cooled down to 40-50°C. The pH of the reaction liquid was adjusted to 6-7 and the mixture was concentrated. 2-Me-THF (50 mL) was added to the residue, and then the pH was adjusted to 2-3 with 1 N HCl. The aqueous phase and the organic phase were separated, and the organic phase was concentrated. To the residue was added n-heptane to precipitate a solid, which was dried under vacuum to yield 55 g of **Boc-Pharmacophore 2** with a yield of 76.5% as a white solid. ^{1}**H NMR** (400 MHz, $CDCl_3$) δ 6.37 -6.32 (m, 2H), 3.58 (t, J = 5.3 Hz, 4H), 3.32 (t, J = 5.3 Hz, 4H), 1.49 (s, 9H). ^{13}**C NMR** (151 MHz, $CDCl_3$) δ 166.39, 164.76 (J = 9.0 Hz), 163.06 (J = 9.0 Hz), 154.43 (J = 58.5 Hz), 97.95, 97.32 (J = 3.0 Hz), 97.14, 80.58, 46.62, 28.39. **HRMS** (ESI) m/z calcd. for $C_{16}H_{20}F_2N_2O_4Na^+$ $[M+Na]^+$: 365.1283, found 365.1288.

Procedure for the synthesis of tert-butyl 4-(4-((4-(2-(4-chlorophenyl)but-3-yn-2-yl)thiazol-2-yl)carbamoyl)-3,5-difluorophenyl)piperazine-1-carboxylate (VIII)

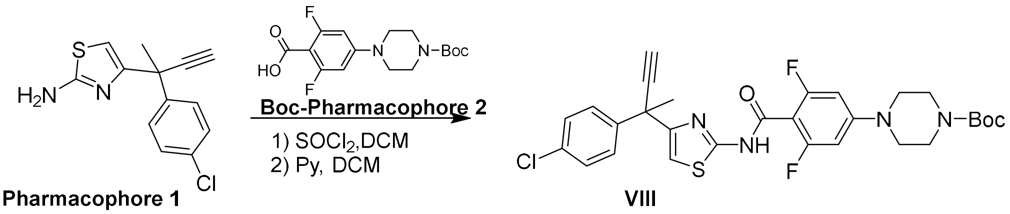

In a five-batch process, to a solution of 4-(4-(tert-butoxycarbonyl)piperazin-1-yl)-2,6-difluorobenzoic acid (**Boc-Pharmacophore 2)** (3.79 g, 11.07 mmol) in DCM (150 mL), $SOCl_2$ (11.07 mmol, 803.12 uL) and DMF (1.30 mmol, 100 μL) were added. The mixture was stirred at 25°C for 1 h. Subsequently, pyridine (55.30 mmol, 4.46 mL) was added and the reaction was stirred at 25°C for 5 min, 4-(4-(tert-butoxycarbonyl)piperazin-1-yl)-2,6-difluorobenzoic acid **(Pharmacophore 1)** (2.76 g, 10.51 mmol) was then added and the mixture was stirred at 25°C for 16 h. The reaction mixture was concentrated under reduced pressure to give a residue. The residue was purified by column chromatography (PE:EA=1:0 to 2:1) to give compound **VIII** (19.9 g combined from 5 batches, yield: 64.5%) as a light yellow solid. ^{1}**H**

NMR (600 MHz, $CDCl_3$) δ 9.45 (br s, 1H), 7.44-7.41 (m, 2H), 7.28-7.25 (m, 2H), 6.92 (s, 1H), 6.38 (dd, J = 13.3, 5.7 Hz, 2H), 3.59-3.56 (m, 4H), 3.33-3.31 (m, 4H), 2.57 (s, 1H), 1.95 (s, 3H), 1.48 (s, 9H). ^{13}**C NMR** (151 MHz, $CDCl_3$) δ 163.49, 161.82, 158.04, 157.67, 154.48, 154.00, 142.64, 132.76, 128.40, 128.38, 128.05, 109.39, 97.46, 97.26, 87.59, 80.48, 72.48, 46.61, 42.58, 29.60, 28.39. **HRMS** (ESI) m/z calcd. for $C_{29}H_{29}ClF_2N_4O_3SNa^+$ $[M+Na]^+$: 609.1509, found 609.1507.

Chiral separation to give Tert-butyl (S)-4-(4-((4-(2-(4-chlorophenyl)but-3-yn-2-yl)thiazol-2-yl)carbamoyl)-3,5-difluorophenyl)piperazine-1-carboxylate (2B)

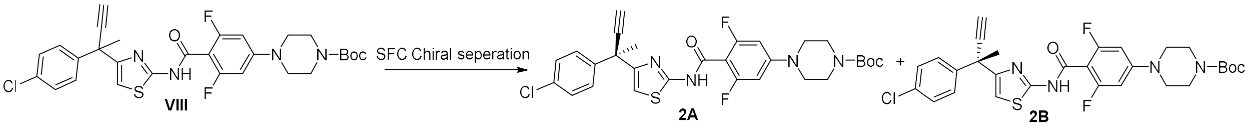

Tert-butyl 4-(4-((4-(2-(4-chlorophenyl)but-3-yn-2-yl)thiazol-2-yl)carbamoyl)-3,5-difluorophenyl)piperazine-1-carboxylate (compound **VIII**, 19.9g, 33.9 mmol) was separated by SFC (column: DAICEL CHIRALCEL OJ (250 mm * 50 mm, 10 um); mobile phase: A: CO_2, B: [0.1%NH_3H_2O in IPA, gradient 40%], back pressure: 100 bar; column temperature: 35°C; Wavelength: 220 nm), to produce two enantiomers. Compound **2A** (8.02 g, yield: 40.3%) and compound **2B** (9.05 g, yield: 45.5%) were obtained as a light yellow solid. Compound **2A** had a retention time of 2.2 min and compound **2B** had a retention time of 4.26 min under the following analytical condition: Column: ChiralCel OJ-H 150 x 4.6 mm, 5 um; Mobile phase: A: CO_2, B: ethanol (0.05% DEA); Isocratic: 48% B; Flow rate: 2.5 ml/min; Column temperature: 40°C; ABPR: 100 bar.

Procedure for the synthesis of compound DF-003

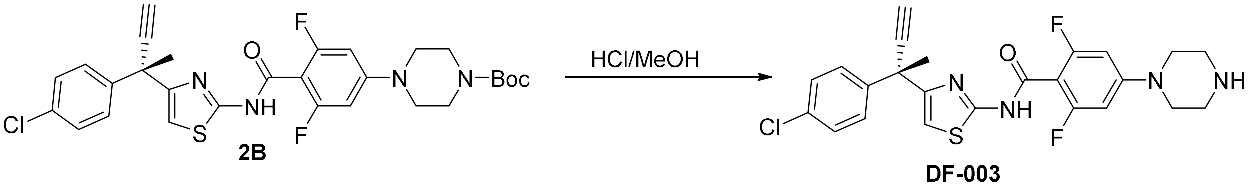

A solution of compound **2B** (9.05 g, 15.42 mmol) in HCl/MeOH (4 M, 150 mL) was stirred at 25°C for 1 h. The reaction mixture was concentrated under reduced pressure. The residue was then dissolved in acetonitrile (50 mL) and concentrated under reduced pressure to give compound **DF-003** (7.28 g, purity 99.7%, yield: 97.0%) as a white solid. The HPLC analytical conditions were as follows: Agilent 1260 HPLC with UV detector and a detection wavelength of 220 nm; Column: Xbridge Shield RP18 (150 mm * 4.6 mm, 3.5 μm); Column Temperature: 45ºC; Mobile Phase A:10 mM NH4OAc in Water; Mobile Phase B: ACN:MeOH =80%:20% (5%-90%).

^{1}**H NMR**(400 MHz, DMSO-d_6) δ 7.46-7.42 (m, 2H), 7.40-7.36 (m, 2H), 7.17 (s, 1H), 6.63 (d, J = 12.8 Hz, 2H), 3.51 (s, 1H), 3.21-3.18 (m, 4H), 2.79-2.76 (m, 4H), 1.91 (s, 3H). ^{13}**C NMR** (101 MHz, DMSO-d_6) 160.76 (dd, J=246.0, 10.7 Hz), 159.58, 159.04, 158.07, 153.83 (t, J=13.6 Hz), 143.29, 131.42, 128.23, 128.14, 108.82, 100.81 (t, J=20.7 Hz), 96.37 (d, J=28.0 Hz), 87.48, 75.12, 47.56, 45.11, 42.17, 29.21. **HRMS** (ESI) m/z calcd. for $C_{24}H_{22}ClF_2N_4OS^+$ [M+H]$^+$: 487.1165, found 487.1172.

DF-003 crystal growth for single crystal X-ray analysis

DF-003 was recrystallized from ethyl acetate (EA) as a colorless granular crystal. Briefly, 100 mg of DF-003 was weighed into a vial, and 0.5 mL of EA was added to yield a solution. Dichloromethane was added dropwise until visual clouding occurred, and then an extra 3-5 drops of dichloromethane were added. The vial was kept still and open, and allowed to volatilize slowly until granular crystals had formed.

DF-003 crystal structure

The configuration of the chiral carbon in DF-003 was determined to be S. The crystal structure of DF-003 has been deposited in the Cambridge Crystallographic Data Centre (Deposition Number 2403779).

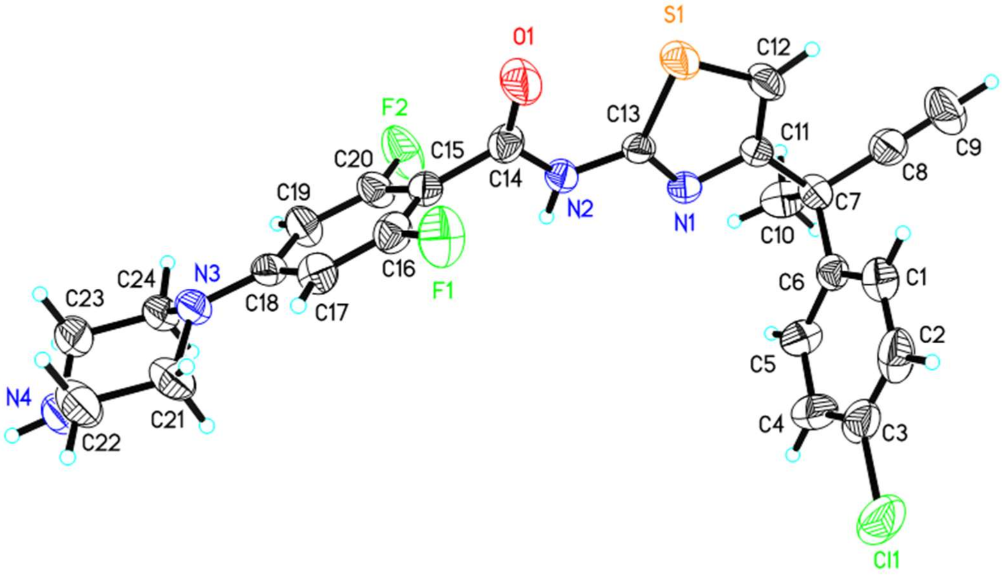

Supplementary Note Figure 5. X-ray structure of DF-003

Supplementary References

1. Yang Y, Ye Q, Jia Z, Cote GP. Characterization of the Catalytic and Nucleotide Binding Properties of the alpha-Kinase Domain of Dictyostelium Myosin-II Heavy Chain Kinase A. *J Biol Chem*. 2015;290:23935-23946. doi: 10.1074/jbc.M115.672410
2. Middelbeek J, Clark K, Venselaar H, Huynen MA, van Leeuwen FN. The alpha-kinase family: an exceptional branch on the protein kinase tree. *Cell Mol Life Sci*. 2010;67:875-890. doi: 10.1007/s00018-009-0215-z
3. Crawley, S.W., Gharaei, M.S., Ye, Q., Yang, Y., Raveh, B., London, N., Schueler-Furman, O., Jia, Z. and Côté, G.P., 2011. Autophosphorylation activates Dictyostelium myosin II heavy chain kinase A by providing a ligand for an allosteric binding site in the α-kinase domain. *Journal of Biological Chemistry*, *286*(4), pp.2607-2616.
4. Henary, E., Casa, S., Dost, T.L., Sloop, J.C. and Henary, M., 2024. The Role of Small Molecules Containing Fluorine Atoms in Medicine and Imaging Applications. *Pharmaceuticals*, *17*(3), p.281.
