# Supplementary material for: Discovery of a selective alpha-kinase 1 inhibitor for the rare genetic disease ROSAH syndrome
Source: Nat Commun. 2025 Sep 9;16:8251. doi: 10.1038/s41467-025-63731-5 (PMC12420824; doi:10.1038/s41467-025-63731-5)
Supplement: Supplementary file 2 — Description of Addtional Supplementary Files [file 41467_2025_63731_MOESM2_ESM.docx]

Supplementary Data 1:

Input, parameters, and output files of molecular docking and molecular dynamics simulations.

Supplementary Data 2:

Inhibitory activity of 10 µM DF-003 against 394 human kinases.
