# Supplementary material for: Discovery of a selective alpha-kinase 1 inhibitor for the rare genetic disease ROSAH syndrome
Source: Nat Commun. 2025 Sep 9;16:8251. doi: 10.1038/s41467-025-63731-5 (PMC12420824; doi:10.1038/s41467-025-63731-5)
Supplement: Supplementary file 5 — Reporting Summary [file 41467_2025_63731_MOESM5_ESM.pdf]

nature portfolio

Corresponding author(s): Cong Xu
Last updated by author(s): Aug 1, 2025

Reporting Summary

Nature Portfolio wishes to improve the reproducibility of the work that we publish. This form provides structure for consistency and transparency in reporting. For further information on Nature Portfolio policies, see our Editorial Policies and the Editorial Policy Checklist.

Statistics

For all statistical analyses, confirm that the following items are present in the figure legend, table legend, main text, or Methods section.

n/a	Confirmed	
☐	☒	The exact sample size (*n*) for each experimental group/condition, given as a discrete number and unit of measurement
☐	☒	A statement on whether measurements were taken from distinct samples or whether the same sample was measured repeatedly
☐	☒	The statistical test(s) used AND whether they are one- or two-sided<br>*Only common tests should be described solely by name; describe more complex techniques in the Methods section.*
☒	☐	A description of all covariates tested
☐	☒	A description of any assumptions or corrections, such as tests of normality and adjustment for multiple comparisons
☐	☒	A full description of the statistical parameters including central tendency (e.g. means) or other basic estimates (e.g. regression coefficient) AND variation (e.g. standard deviation) or associated estimates of uncertainty (e.g. confidence intervals)
☐	☒	For null hypothesis testing, the test statistic (e.g. *F*, *t*, *r*) with confidence intervals, effect sizes, degrees of freedom and *P* value noted<br>*Give P values as exact values whenever suitable.*
☒	☐	For Bayesian analysis, information on the choice of priors and Markov chain Monte Carlo settings
☒	☐	For hierarchical and complex designs, identification of the appropriate level for tests and full reporting of outcomes
☒	☐	Estimates of effect sizes (e.g. Cohen's *d*, Pearson's *r*), indicating how they were calculated

Our web collection on statistics for biologists contains articles on many of the points above.

Software and code

Policy information about availability of computer code

Data collection	Applied Biosystems Quantstudio 5 for qRT-PCR; Skanlt Software 5.0 for Microplate Readers RE, ver. 5.0.0.42 for ELISA; Motic Images Plus 3.0 for light and fluorescent microscopy images acquisition
Data analysis	Statistical analyses were performed using GraphPad Prism sofrtware (GraphPad Software, Inc.). For protein crystallography data processing, DENZO and SCALEPACK (HKL Research Inc.), Coot (MRC Laboratory of Molecular Biology) and PHENIX (QAD Inc.) were used. For molecular docking analysis, Maestro (Schrödinger Suites 2021-2), Glide (Schrödinger Suites 2021-2) and Prime (Schrödinger Suites 2021-2) were used. For SPR experiment, Biacore Insight Evaluation software version 5 (Cytiva) was used.

For manuscripts utilizing custom algorithms or software that are central to the research but not yet described in published literature, software must be made available to editors and reviewers. We strongly encourage code deposition in a community repository (e.g. GitHub). See the Nature Portfolio guidelines for submitting code & software for further information.

Data

Policy information about availability of data

All manuscripts must include a data availability statement. This statement should provide the following information, where applicable:
- Accession codes, unique identifiers, or web links for publicly available datasets
- A description of any restrictions on data availability
- For clinical datasets or third party data, please ensure that the statement adheres to our policy

The crystal structure of DF-003 has been deposited in the Cambridge Crystallographic Data Centre under deposition numbers CCDC 2403779. Copies of the data can

be obtained free of charge via https://www.ccdc.cam.ac.uk/structures/.(Deposition Number 2403779). Macromolecular structural data have been deposited in the Worldwide Protein Data Bank (wwPDB, Deposition ID 9J4P, https://doi.org/10.2210/pdb9J4P/pdb). Input, parameter, and output files of molecular docking and molecular dynamic simulation analyses are provided in Supplementary Data. Source data in EXCEL format for all charts in this study and uncropped Western Blot image are available under "Source Data" in this article. The remaining data are available within the Article, Supplementary data files or the Source Data file provided with this manuscript.

Research involving human participants, their data, or biological material

Policy information about studies with human participants or human data. See also policy information about sex, gender (identity/presentation), and sexual orientation and race, ethnicity and racism.

Reporting on sex and gender	*Use the terms sex (biological attribute) and gender (shaped by social and cultural circumstances) carefully in order to avoid confusing both terms. Indicate if findings apply to only one sex or gender; describe whether sex and gender were considered in study design; whether sex and/or gender was determined based on self-reporting or assigned and methods used. Provide in the source data disaggregated sex and gender data, where this information has been collected, and if consent has been obtained for sharing of individual-level data; provide overall numbers in this Reporting Summary. Please state if this information has not been collected. Report sex- and gender-based analyses where performed, justify reasons for lack of sex- and gender-based analysis.*
Reporting on race, ethnicity, or other socially relevant groupings	*Please specify the socially constructed or socially relevant categorization variable(s) used in your manuscript and explain why they were used. Please note that such variables should not be used as proxies for other socially constructed/relevant variables (for example, race or ethnicity should not be used as a proxy for socioeconomic status). Provide clear definitions of the relevant terms used, how they were provided (by the participants/respondents, the researchers, or third parties), and the method(s) used to classify people into the different categories (e.g. self-report, census or administrative data, social media data, etc.) Please provide details about how you controlled for confounding variables in your analyses.*
Population characteristics	*Describe the covariate-relevant population characteristics of the human research participants (e.g. age, genotypic information, past and current diagnosis and treatment categories). If you filled out the behavioural & social sciences study design questions and have nothing to add here, write "See above."*
Recruitment	*Describe how participants were recruited. Outline any potential self-selection bias or other biases that may be present and how these are likely to impact results.*
Ethics oversight	*Identify the organization(s) that approved the study protocol.*

Note that full information on the approval of the study protocol must also be provided in the manuscript.

Field-specific reporting

Please select the one below that is the best fit for your research. If you are not sure, read the appropriate sections before making your selection.

☒ Life sciences ☐ Behavioural & social sciences ☐ Ecological, evolutionary & environmental sciences

For a reference copy of the document with all sections, see nature.com/documents/nr-reporting-summary-flat.pdf

Life sciences study design

All studies must disclose on these points even when the disclosure is negative.

Sample size	No statistical method was used to predetermine sample size. The sample size was determined based on previous experience of reproducibility with similar experimental models while minimizing laboratory animal use.
Data exclusions	Statistical outliers were determined by the Grubb's test and indicated in the source data file. Occasionally for immunohistochemistry and qRT-PCR analyses, as indicated in the figure legend, samples were excluded due to unsuccessful processing of the material.
Replication	All experiments have been successfully repeated with similar results at least once.
Randomization	In in vitro studies, technical triplicate or quadruplicate wells were not randomized.<br>In animal studies, among mice of the same genotype, each mouse was first assigned a random number provided by EXCEL (Microsoft), then ordered and grouped to each treatment group. Minor adjustment might be applied to ensure animals of each treatment group have same average body weight.
Blinding	Investigators were blinded to genotype and treatment conditions during acquisition and analyses of retinal immunofluorescent/ immunohistochemistry images, visual acuity measurement, fundus imaging, OCT imaging, anhidrosis test and multiplex serum chemokine measurements of animals. Blinding was not performed on crystallography, homology modeling and molecular docking, in vitro kinase assays, qRT-PCR, ELISA, LC-MS/MS in phamacokinetic analyses, body weight and organ weight measurement of animals because data collection for these studies is automated and confers high objectivity.

Reporting for specific materials, systems and methods

We require information from authors about some types of materials, experimental systems and methods used in many studies. Here, indicate whether each material, system or method listed is relevant to your study. If you are not sure if a list item applies to your research, read the appropriate section before selecting a response.

Materials & experimental systems

n/a	Involved in the study
☐	☒ Antibodies
☐	☒ Eukaryotic cell lines
☒	☐ Palaeontology and archaeology
☐	☒ Animals and other organisms
☒	☐ Clinical data
☒	☐ Dual use research of concern
☒	☐ Plants

Methods

n/a	Involved in the study
☒	☐ ChIP-seq
☒	☐ Flow cytometry
☒	☐ MRI-based neuroimaging

Antibodies

Antibodies used	Iba1 (Wako, 019-19741)<br>GFAP (Abcam; ab68428)
Validation	All antibodies are commercial in origin. Validation statements found on the manufacturer's website for the following:<br>Iba1: https://labchem-wako.fujifilm.com/us/product/detail/W01W0101-1974.html<br>GFAP: https://www.abcam.com/en-us/products/primary-antibodies/gfap-antibody-epr1034y-ab68428

Eukaryotic cell lines

Policy information about cell lines and Sex and Gender in Research

Cell line source(s)	HEK293 and THP-1 cells were obtained from the Cell Bank of The Chinese Academy of Sciences
Authentication	Identity of the cell lines were frequently checked by their morphology but have not been authenticated by the short tandem repeat (STR profiling)
Mycoplasma contamination	All cells were tested to be mycoplasma-negative by the standard PCR method.
Commonly misidentified lines (See ICLAC register)	No commonly misidentified cell lines were used.

Animals and other research organisms

Policy information about studies involving animals; ARRIVE guidelines recommended for reporting animal research, and Sex and Gender in Research

Laboratory animals	Pharmacokinetic analyses: 8-week-old male wildtype C57BL/6J mice<br>In vivo efficacy test of the compound: 16-17-week-old female C57BL/6J mice<br>Splenomegaly and anhidrosis test: 4-month- and 9-month- old male C57BL/6J mice<br>Fundus imaging, OCT imaging, and assessment of visual acuity: 4-month- and 9-month-old female C57BL/6J mice<br>In all above analyses except for pharmacokinetic study, mice of two genotypes, hALPK1-KI and hALPK1[T237M]-KI, are involved
Wild animals	Current study did not involve wild animals.
Reporting on sex	Phamacokinetic analyses, splenomegaly and anhidrosis test were also performed in female mice with similar findings.<br>In vivo efficacy test was also performed in male mice with similar findings as in female mice.<br>Due large group sizes and samples sizes, female mice and male mice were tested in independent experiments and samples analyzed separately.
Field-collected samples	Current study did not involve field-collected samples.
Ethics oversight	All animal studies were approved by the Institutional Animal Care and Use Committees (IACUC) at Shanghai Yao Yuan Biotechnology Ltd. (Drug Farm), Zhejiang Yao Yuan Biotechnology Ltd. and Oujiang Laboratory in accordance with the national guidelines for housing and care of laboratory animals (Ministry of Health, Beijing, China).

Note that full information on the approval of the study protocol must also be provided in the manuscript.

Plants

Seed stocks	*Report on the source of all seed stocks or other plant material used. If applicable, state the seed stock centre and catalogue number. If plant specimens were collected from the field, describe the collection location, date and sampling procedures.*
Novel plant genotypes	*Describe the methods by which all novel plant genotypes were produced. This includes those generated by transgenic approaches, gene editing, chemical/radiation-based mutagenesis and hybridization. For transgenic lines, describe the transformation method, the number of independent lines analyzed and the generation upon which experiments were performed. For gene-edited lines, describe the editor used, the endogenous sequence targeted for editing, the targeting guide RNA sequence (if applicable) and how the editor was applied.*
Authentication	*Describe any authentication procedures for each seed stock used or novel genotype generated. Describe any experiments used to assess the effect of a mutation and, where applicable, how potential secondary effects (e.g. second site T-DNA insertions, mosiacism, off-target gene editing) were examined.*
